# Skeletal Muscle HSF1 Alleviates Age‐Associated Sarcopenia and Mitochondrial Function Decline via SIRT3‐PGC1α Axis

**DOI:** 10.1002/advs.202510368

**Published:** 2025-12-16

**Authors:** Jun Zhang, Min Hu, Xia Wu, Mingwei Guo, Ying Ma, Jin Qiu, Siqi Wang, Yuxiang Cao, Yinzhao Zhong, Fangfang Chen, Yiwen Wang, Wei Wei, Yan Lu, Yong Zhang, Junjie Xiao, Zhenji Gan, Cheng Hu, Xinran Ma, Lingyan Xu

**Affiliations:** ^1^ Shanghai Key Laboratory of Regulatory Biology Institute of Biomedical Sciences and School of Life Sciences East China Normal University Shanghai 200241 China; ^2^ Shanghai Diabetes Institute Shanghai Key Laboratory of Diabetes Mellitus Shanghai Clinical Centre for Diabetes Shanghai Jiao Tong University Affiliated Sixth People's Hospital Shanghai 200233 China; ^3^ Institute of Metabolism and Regenerative Medicine Digestive Endoscopic Center Shanghai Sixth People's Hospital Affiliated to Shanghai Jiao Tong University School of Medicine Shanghai 200233 China; ^4^ State Key Laboratory for Complex Severe, and Rare Diseases Institute of Basic Medical Sciences Chinese Academy of Medical Sciences and School of Basic Medicine Peking Union Medical College Beijing 100005 China; ^5^ Bioland Laboratory (Guangzhou Regenerative Medicine and Health Guangdong Laboratory) Guangzhou 510005 China; ^6^ Cardiac Regeneration and Ageing Lab Institute of Cardiovascular Sciences Shanghai Engineering Research Center of Organ Repair School of Life Sciences Shanghai University Shanghai 200444 China; ^7^ State Key Laboratory of Pharmaceutical Biotechnology and MOE Key Laboratory of Model Animal for Disease Study Model Animal Research Center Medical School of Nanjing University Nanjing 210093 China; ^8^ Department of Endocrinology and Metabolism Fengxian Central Hospital Affiliated to Southern Medical University Shanghai 201499 China; ^9^ Shanghai Frontiers Science Center of Genome Editing and Cell Therapy Shanghai Key Laboratory of Regulatory Biology and School of Life Sciences East China Normal University Shanghai 200241 China; ^10^ Institute for Aging East China Normal University Shanghai 200241 China

**Keywords:** aging, mitochondria, muscle atrophy, oxidative function, sarcopenia

## Abstract

Age‐related sarcopenia, characterized by progressive loss of skeletal muscle mass and strength, impacts metabolic health and quality of life in the elderly. Heat shock factor 1 (HSF1) is a transcription factor that orchestrates cellular responses to various stresses, while its role in sarcopenia remains unknown. Here, *HSF1* mRNA expression was decreased in muscles of aged mice and humans, correlating negatively with the atrophic gene and positively with the mitochondrial gene. Aged HSF1 muscle‐specific knockout mice exhibited severe muscle atrophy and reduced endurance capacity, partially due to smaller fast fibers and mitochondrial dysfunction in slow fibers, as well as impaired systemic metabolic performance. In contrast, HSF1 overexpression in skeletal muscle improved these functions. Mechanistically, via RNA sequencing (RNA‐seq) and chromatin immunoprecipitation sequencing (ChIP‐seq), it is revealed that HSF1 transcriptionally activated Sirtuin3 (SIRT3) for the deacetylation of both PGC1α1 and PGC1α4 isoforms of peroxisome proliferator‐activated receptor gamma coactivator 1‐alpha (PGC1α), in skeletal muscle, enhancing mitochondrial function and muscle hypertrophy in vivo and in vitro, and inducing fibronectin type III domain‐containing protein 5 (FNDC5)/Irisin for tissue crosstalk. Thus, HSF1 regulates skeletal muscle functions and systemic energy homeostasis via the SIRT3‐PGC1α axis, representing a potential therapeutic target for sarcopenia and metabolic disorders.

## Graphical abstract

Aged HSF1 muscle‐specific knockout mice show deteriorated muscle atrophy and metabolic dysfunction, while active HSF1 overexpression improves muscle function via activating SIRT3 to deacetylate both PGC1α1 and PGC1α4, which boosts mitochondrial function and muscle hypertrophy in a fiber‐type specific manner, and induces FNDC5/Irisin for tissue crosstalk, suggesting HSF1 as a potential therapeutic target for age‐related sarcopenia and metabolic disorders.

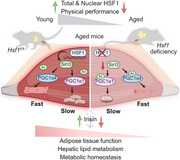

## 1 Introduction

Skeletal muscle accounts for ≈ 40% of body mass and stands as a pivotal hub for metabolic activities and energy homeostasis.^[^ ^1^ ^]^ Myofibers, the basic constituent units of skeletal muscle, are a heterogeneous population based on distinct myosin composition, contractile ability, and metabolic profiles.^[^ ^2^ ^]^ Specifically, myofibers can be categorized into slow‐twitch oxidative (type I) and fast‐twitch (type II) fibers, each with unique characteristics. Slow‐twitch oxidative (type I) fibers feature high mitochondrial density and robust oxidative capacity, which can meet the enhanced energy demands during prolonged physical activity and are thus important for long‐term endurance performance. In contrast, fast‐twitch (type II) fibers can be further divided into two subtypes based on metabolic heterogeneity: fast oxidative (type IIa) and fast glycolytic (type IIx/b) subtypes. Fast glycolytic (type IIx/b) fibers are larger in size, less mitochondrial‐enriched, and exhibit a prominent glycolytic capacity, making them suitable for generating powerful and short bursts of movement, while fast oxidative glycolytic (type IIa) fibers possess intermediate properties. Both fast‐ and slow‐twitch muscle fibers contribute to energy homeostasis as well as glucose and lipid metabolism through finely‐tuned mitochondrial biogenesis and function.^[^ ^3^ ^]^

Skeletal muscle undergoes age‐related sarcopenia, a progressive and generalized loss of skeletal muscle mass and function during aging, often accompanied with a progressive increase in body fat and subsequent systemic metabolic dysregulation, including insulin resistance and fatty liver.^[^ ^4^ ^]^ The loss of proteostasis and mitochondrial dysfunction are both important for sarcopenia.^[^ ^4^ ^]^ The imbalance between muscle anabolic and catabolic pathways results in a net loss of muscle proteins, organelles and cytoplasm, eventually leading to loss of skeletal muscle.^[^ ^5^ ^]^ Besides, mitochondrial dysfunctions, evidenced by impaired oxidative phosphorylation and mitochondrial biogenesis, also contribute to the decline in muscle function.^[^ ^6^ ^]^ Improvements in mitochondrial respiration and biogenesis of skeletal muscle can alleviate age‐related sarcopenia.^[^ ^7^ ^]^ Interestingly, fast fibers are more susceptible to aging‐related atrophy. In contrast, slow fibers have more ribosomes and higher expression of proteasomes and chaperones to support protein turnover and maintain their mass, but are more prone to reduced oxidative enzyme activities and respiratory capacity during aging.^[^ ^8^ ^]^ Thus, the multifaceted nature of sarcopenia poses challenges in identifying a key regulator that could effectively improve both types of muscle fibers during aging.

Heat shock transcription factor 1 (HSF1) is a transcription factor that coordinates the cellular response to proteotoxic stress by activating the transcription of heat shock proteins (HSPs). It has been well established that HSF1 promotes longevity and multiple tissue homeostasis via protein quality control, stress adaptation and cell survival.^[^ ^9^ ^]^ Recently, we and others have discovered the roles and mechanisms of HSF1 in metabolic control, including browning of white fat, insulin sensitivity and hepatic steatosis.^[^ ^10^, ^11^, ^12^ ^]^ Besides, HSF1 has been shown to be involved in muscle atrophy and regeneration in mouse models with hindlimb suspension, synergist muscle ablation, or intramuscular injection of cardiotoxin (CTX), possibly via the induction of heat shock proteins and suppression of proinflammatory cytokines, such as Interleukin‐6 (IL‐6).^[^ ^13^, ^14^, ^15^ ^]^ Of note, in addition to classic HSP activation, we and others have identified numerous unconventional HSF1 target genes via chromatin immunoprecipitation sequencing (ChIP‐seq) in different systems.^[^ ^12^, ^16^, ^17^ ^]^ Specifically, we revealed the transcriptional and post‐transcriptional molecular links between HSF1 and peroxisome proliferator‐activated receptor gamma coactivator 1‐alpha (PGC1α), a key regulator of energy metabolism, mitochondrial biogenesis, and function in both skeletal muscle and thermogenic fat. In addition, it has been reported that PGC1α1 and PGC1α4, two key isoforms of PGC1α, regulate mitochondrial functions and muscle hypertrophy in skeletal muscle via different mechanisms.^[^ ^18^ ^]^ Considering that age‐related muscle atrophy and subsequent metabolic dysregulation impact wide populations, we decided to investigate the roles of HSF1 during aging in both myofiber atrophy and mitochondrial dysfunction in muscle, as well as its contribution to systemic energy metabolism.

In the present study, we investigated the changes and roles of HSF1 in skeletal muscle during aging. By assessing metabolic phenotypes of muscle HSF1 loss‐ and gain‐of‐function models, we revealed the important protective roles of HSF1 in age‐associated sarcopenia and metabolic disorders. Mechanistically, we utilized ChIP‐seq and RNA‐seq to understand the unconventional HSF1 target in muscle and revealed that HSF1 regulated the transcription of Sirtuin 3 (Sirt3), which deacetylated PGC1α1 and PGC1α4 to prevent muscle atrophy and improve mitochondrial function. In addition, a reported PGC1α downstream target, fibronectin type III domain‐containing protein 5 (FNDC5)/Irisin, was also elevated and mediated possible tissue crosstalk between muscle and adipose tissues. Thus, our study proposed HSF1 as a potential therapeutic target against sarcopenia and aging‐related metabolic dysfunction.

## 2 Results

### 2.1 Skeletal Muscle HSF1 Levels are Reduced During Aging and Correlate with Muscle Atrophic and Mitochondrial Gene Expression in Mice and Humans

**Figure 1:**
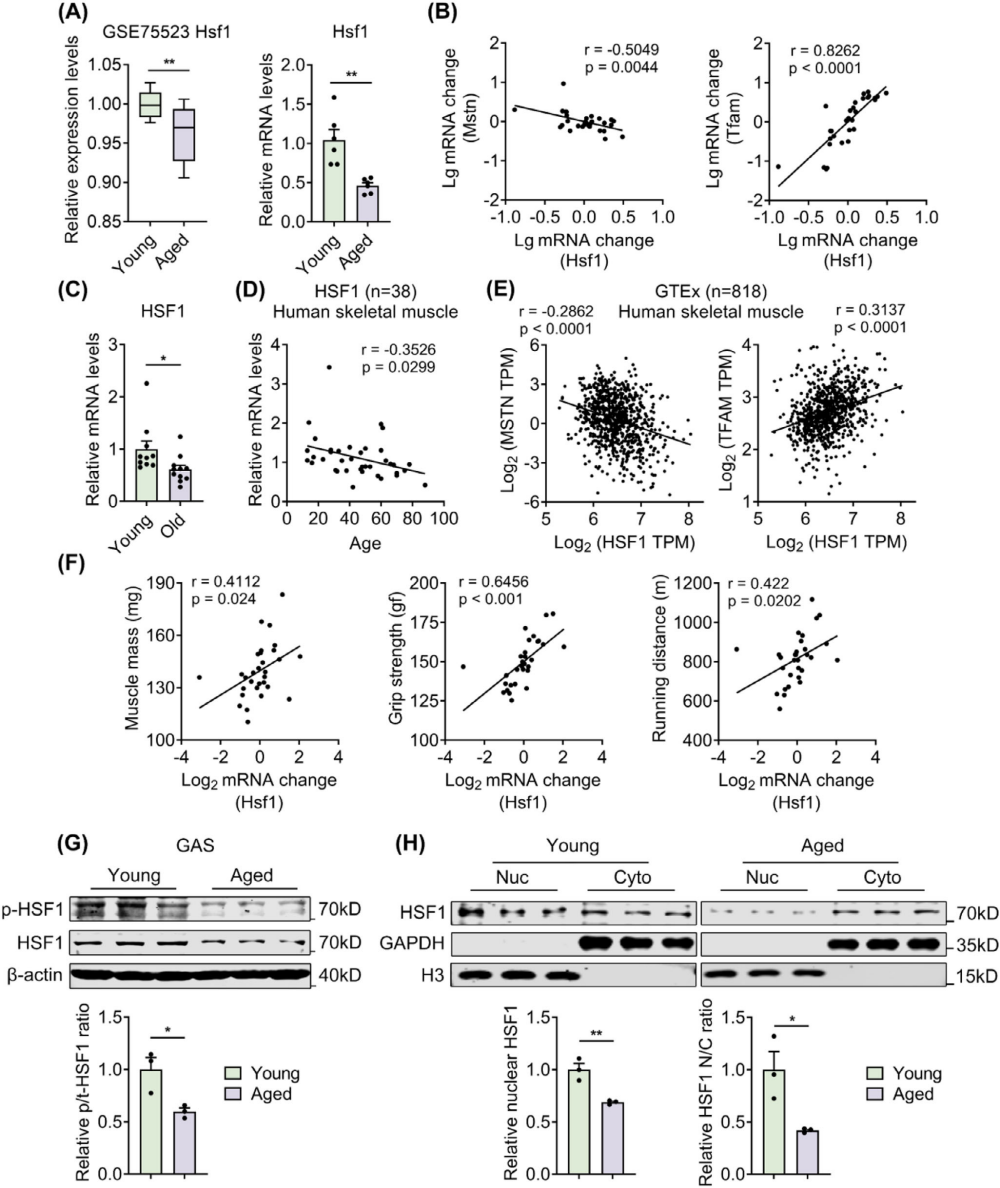
Skeletal muscle HSF1 levels are reduced during aging and correlate with genes related to muscle atrophic and mitochondrial genes in mice and humans. A) *Hsf1* mRNA levels in the hind limb muscle of young and aged mice from GSE75523 (left, n = 16) and *Hsf1* mRNA levels (qRT‐PCR) in GAS muscles of young and aged mice (right, n = 6). B) Pearson correlation analysis between *Hsf1* and *Mstn* or *Tfam* mRNA levels (qRT‐PCR) in GAS of mice (n = 30). C) *HSF1* mRNA levels (qRT‐PCR) in vastus lateralis muscle biopsies from young and old individuals (n = 10‐11). D) Pearson correlation analysis between *HSF1* mRNA levels (qRT‐PCR) in human muscles and age (n = 38). E) Pearson correlation analysis between *HSF1* and *MSTN* or *TFAM* mRNA levels obtained from GTEx human muscles (right, n = 818). F) Pearson correlation analysis of *Hsf1* mRNA levels with muscle mass, grip strength, and running endurance in mice (n = 30). G) Immunoblotting analysis and quantification of HSF1 phosphorylation at Ser326 (p‐HSF1) and total HSF1 in GAS muscles from young or aged mice (n = 3). H) Immunoblotting analysis of HSF1 in nuclear (Nuc) and cytoplasmic (Cyto) fractions from young or aged GAS muscle, with quantification of nuclear HSF1 levels and Nuc/Cyto ratio. GAPDH and Histone H3 served as cytoplasmic and nuclear controls, respectively. Statistical significance was assessed by unpaired Student's *t*‐test (A, C, and F‐I) and Pearson's r correlation coefficient (B, D and E). Data are presented as mean ± SEM and *p < 0.05; **p < 0.01; ns, not significant compared to control group.

To elucidate the role of Heat shock factor 1 (HSF1) in muscle aging, we first analyzed the gene expression levels of *Hsf1* in muscles from young and aged mice. The results from the GEO database and our in‐house samples both showed that *Hsf1* mRNA levels were significantly reduced in the hindlimb (GSE75523) and gastrocnemius (GAS) muscles of aged mice compared to those of young mice (Figure **1**A). Besides, *HSF1* mRNA levels were negatively correlated with myostatin (*Mstn)*, a muscle atrophic gene that is linked to muscle atrophy, while positively correlated with transcription factor A, mitochondrial (*Tfam)*, a mitochondrial gene associated with mitochondrial biogenesis and function (Figure 1B).^[^ ^19^, ^20^ ^]^ To determine the clinical relevance of *HSF1* mRNA expression levels in human skeletal muscle in the context of aging, we measured *HSF1* mRNA levels in vastus lateralis muscle biopsies from human volunteers. The results showed reduced *HSF1* levels in muscles from old subjects (age>60) than those from young volunteers (age < 30) (Figure 1C). Pearson correlation analysis demonstrated a significant negative correlation between *HSF1* mRNA levels and age (Figure 1D). Besides, *HSF1* mRNA levels were also negatively correlated with *MSTN*, while positively correlated with *TFAM* in human samples from the Genotype‐Tissue Expression (GTEx) database (Figure 1E). To further assess the physiological relevance of HSF1, we performed correlation analysis and found that *Hsf1* mRNA levels were significantly and positively correlated with muscle mass, grip strength, and treadmill endurance in muscle (Figure 1F). To provide deeper insight into compromised HSF1 function during aging, we have performed western blot analysis to assess HSF1 activation via HSF1 Ser326 phosphorylation^[^ ^9^ ^]^ and HSF1 subcellular localization in GAS muscle of young and aged mice. Consistent with our mRNA data, total HSF1 protein levels showed marked reduction in aged muscle. Interestingly, we found reduced p‐HSF1/t‐HSF1 ratio and impaired HSF1 nuclear localization in aged muscle, suggesting that HSF1 transactivation capacity was diminished in muscle of aged mice (Figure 1G,H). The phenotype is similar to our recent report that peroxisome proliferator‐activated receptor gamma coactivator 1‐alpha isoform 4 (PGC1α4), the downstream effector of HSF1, exhibited defects in expression and nuclear localization in aging,^[^ ^21^ ^]^ suggesting the dual mechanisms for the compromised functions of HSF1‐PGC1α4 during age‐associated sarcopenia. Overall, these data suggested that HSF1 levels were decreased in skeletal muscle during aging, and it was closely correlated with muscle atrophy and mitochondrial function.

### 2.2 Muscle HSF1 Deficiency Exacerbates Age‐Associated Sarcopenia in Aged Mice

To directly examine the roles of HSF1 in skeletal muscle, we generated muscle‐specific HSF1 knockout (HSF1‐MKO) mice under the control of the muscle creatine kinase (MCK) promoter (Figure S1A, Supporting Information). HSF1‐MKO mice have a specific decrease in HSF1 protein levels in muscles, but not in the heart and other tissues, compared to WT littermates (Figure S1B,C, Supporting Information). Interestingly, we found a significant decline in *Hsf1* mRNA levels in cardiac tissue of HSF1‐MKO mice (Figure S1D, Supporting Information), which was consistent with previous report.^[^ ^22^ ^]^ However, the mRNA downregulation of *Hsf1* did not cause a significant change in HSF1 protein levels within the heart (Figure S1C, Supporting Information), which was also reported in previous study using MCK‐Cre models,^[^ ^23^ ^]^ possibly due to compensatory effects at the post‐transcriptional level. Consistently, WT and HSF1‐MKO mice of young or old age showed similar cardiac functions under echocardiographic analysis, including ejection fraction (EF) and fractional shortening (FS) (Figure S1E, Supporting Information). Intriguingly, HSF1‐MKO mice exhibited normal muscular phenotypes at both 3 and 12 months of age, including body composition, grip strength, and muscle mass, indicating that HSF1 is dispensable for postnatal development and basal maintenance in adulthood (Figure S1F–I, Supporting Information).

**Figure 2:**
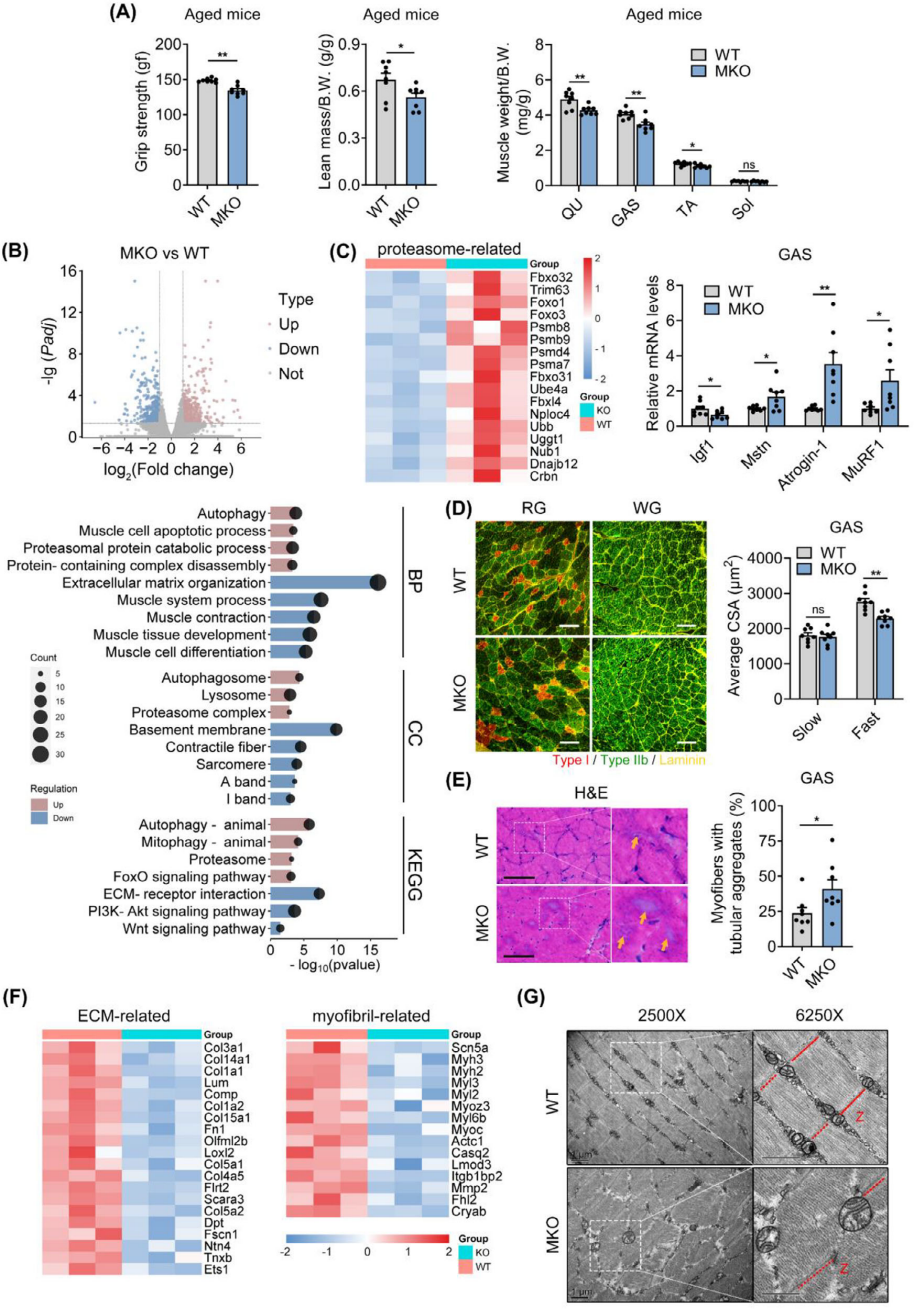
Muscle HSF1 deficiency exacerbates age‐associated sarcopenia in aged mice. A) Whole limb grip strength (left), normalized lean mass (middle), and muscle weights (right) from wild‐type (WT) and *Hsf1^f/f^*;*MCK‐Cre* (HSF1‐MKO) mice at the age of 22 months. B) Volcano plot (Top) showing fold changes versus adjusted *P* values for analyzed RNA‐Seq data generated from the GAS muscles of indicated genotypes. Significantly upregulated genes are represented by red dots, whereas downregulated genes are represented by blue dots. GO and KEGG enrichment analysis of DEGs regulated in MKO muscles (Bottom). C) Heatmap analysis of proteasome‐related genes regulated in MKO muscles compared with WT controls (left) and expression of muscle atrophic genes (qRT‐PCR) in GAS muscles from the indicated genotypes (right). D) Representative MyHC IF staining in GAS muscles of indicated genotypes (left) and quantification of fiber size of slow‐ and fast‐twitch fibers (right). Scale bar represents 100 µm. Red, type I myofibers; black, type IIa/x myofibers; green, type IIb myofibers; yellow, Laminin. E) Representative H&E staining and quantifications of fibers with tubular aggregates in GAS muscles of indicated genotypes. Yellow arrows indicate myofibers with tubular aggregates. Scale bar represents 100 µm. F) Heatmap analysis of extracellular matrix(ECM)‐ and myofibril‐related genes in GAS muscles. G) Representative electron micrographs of muscles showing sarcomere structure and Z‐line in sections of indicated genotypes (right, n = 3). Red dotted line indicates the Z‐line. Scale bar represents 1 µm. n = 8 mice per group unless otherwise noted. Statistical significance was assessed by unpaired Student's *t*‐test (A and C‐E). Data are presented as mean ± SEM and *p < 0.05; **p < 0.01; ns, not significant compared to control group.

Next, we investigated the influences of HSF1 deficiency on the skeletal muscle of aged mice. Individual muscles exhibit distinct fiber‐type compositions, i.e., the tibialis anterior (TA) is predominantly composed of fast‐twitch fibers, the soleus (Sol) muscle of slow‐twitch fibers, while the GAS and the quadriceps femoris (QU) muscles characterized by mixed fiber‐type muscles.^[^ ^24^, ^25^ ^]^ 22‐month‐old HSF1‐MKO mice exhibited reduced grip strength and decreased lean mass compared with WT mice at the same age (**Figure** **2**A). These are mainly contributed by changes in fast‐twitch fibers, as fast‐twitch dominant (TA) and mixed‐type (GAS and QU) muscles showed reduced weights, while slow‐twitch dominant (Sol) muscles had comparable weights (Figure 2A). RNA‐seq analysis was performed in GAS muscles to understand the molecular changes in muscle after HSF1 deficiency (Figure 2B). Gene ontology (GO) and KEGG analysis revealed consistent up‐regulation of genes associated with autophagy and proteasomal protein catabolic process in aged HSF1‐MKO mice (Figure 2B,C), indicative of enhanced protein degradation, a critical event in age‐related muscle atrophy,^[^ ^26^ ^]^ which was confirmed by increased mRNA and protein levels of muscle atrophic genes (Figure 2C; Figure S2A,B, Supporting Information). Besides, we observed decreased muscle hypertrophic mTOR‐S6K1 signaling pathway in GAS and QU muscles of aged HSF1‐MKO mice (Figure S2A,C, Supporting Information). Consistently, we found that fast‐twitch fiber (TA) muscle in aged HSF1‐MKO mice exhibited smaller myofiber sizes compared to aged WT mice (Figure S2D, Supporting Information), while in mixed‐type muscles (GAS and QU), only fast‐twitch fibers (stained positive for MYH4) were smaller in HSF1‐MKO mice, with comparable slow‐twitch fibers (stained positive for MYH7) (Figure 2D; Figure S2E, Supporting Information). Besides, quantification of fiber‐type proportions and total number of myofibers in cross‐sections results demonstrated that HSF1 deficiency did not induce fiber‐type switching, accompanied with the corresponding gene program (Figure S2F, Supporting Information), and not alter the total myofiber number (Figure S2G, Supporting Information). These results suggested that HSF1 deficiency mediated sarcopenia was majorly due to its regulation on the size of individual myofibers, rather than the loss of fiber numbers or fiber‐type switch. Considering that loss of Pax7^+^ satellite cells was known to attribute to sarcopenia progression, we analyzed Pax7^+^ satellite cell numbers in aged muscle of WT and HSF1‐MKO mice. The immunofluorescence data showed no significant changes in satellite cell numbers (Figure S3A, Supporting Information). It is thus possible that muscle specific deficiency of HSF1 may lead to myofiber changes, but not directly affect satellite function. However, we could not fully exclude the possibility that HSF1 mediated changes in myotubes may affect satellite cell functions via cellular crosstalk. Notably, we observed increased age‐dependent occurrence of tubular aggregates as shown by prominent basophilic inclusions in muscles of aged HSF1‐MKO mice (Figure 2E; Figure S2H, Supporting Information).^[^ ^27^ ^]^ Furthermore, RNA‐seq analysis also revealed significant downregulation of genes involved in extracellular matrix (ECM) organization and sarcomeric component‐related terms (Figure 2B,F), and significant enhancement of the interferon‐inflammatory signature in aged HSF1‐MKO muscle (Figure S3B, Supporting Information), which was also reported previously during skeletal muscle aging.^[^ ^28^, ^29^, ^30^, ^31^, ^32^ ^]^ To clarify whether the reduced ECM‐related gene programs in KO mice translate into functional fibrosis benefits, we analyzed Sirius red staining, Pdgfrα^+^ fibro‐adipogenic progenitor (FAP) activity and FAP differentiation genes expression, which showed similar results between WT and HSF1‐MKO mice (Figure S3C–E, Supporting Information). In contrast, muscles of HSF1‐MKO mice featured decreased expressions in various collagen genes, the main components of the extracellular matrix (ECM) (Figure 2F). ECM has been shown to provide critical structural support for sarcomeric anchoring and contractile function.^[^ ^33^ ^]^ Indeed, the ultrastructure of QU muscles under TEM revealed severely abnormal sarcomeric structure, including loss of the characteristic rectangular sarcomere structure and misalignment of Z‐line (Figure 2G), which may contribute to muscle weakness and atrophy in aged HSF1‐MKO mice. These data suggested that the fast‐twitch fibers of aged HSF1‐MKO mice were smaller due to enhanced protein degradation and reduced protein synthesis, which was related to muscle atrophy and disorganized muscle structure.

### 2.3 Muscle HSF1 Deficiency Deteriorates Endurance Capacity and Causes Mitochondrial Dysfunction in Oxidative Fibers of Aged Mice

**Figure 3:**
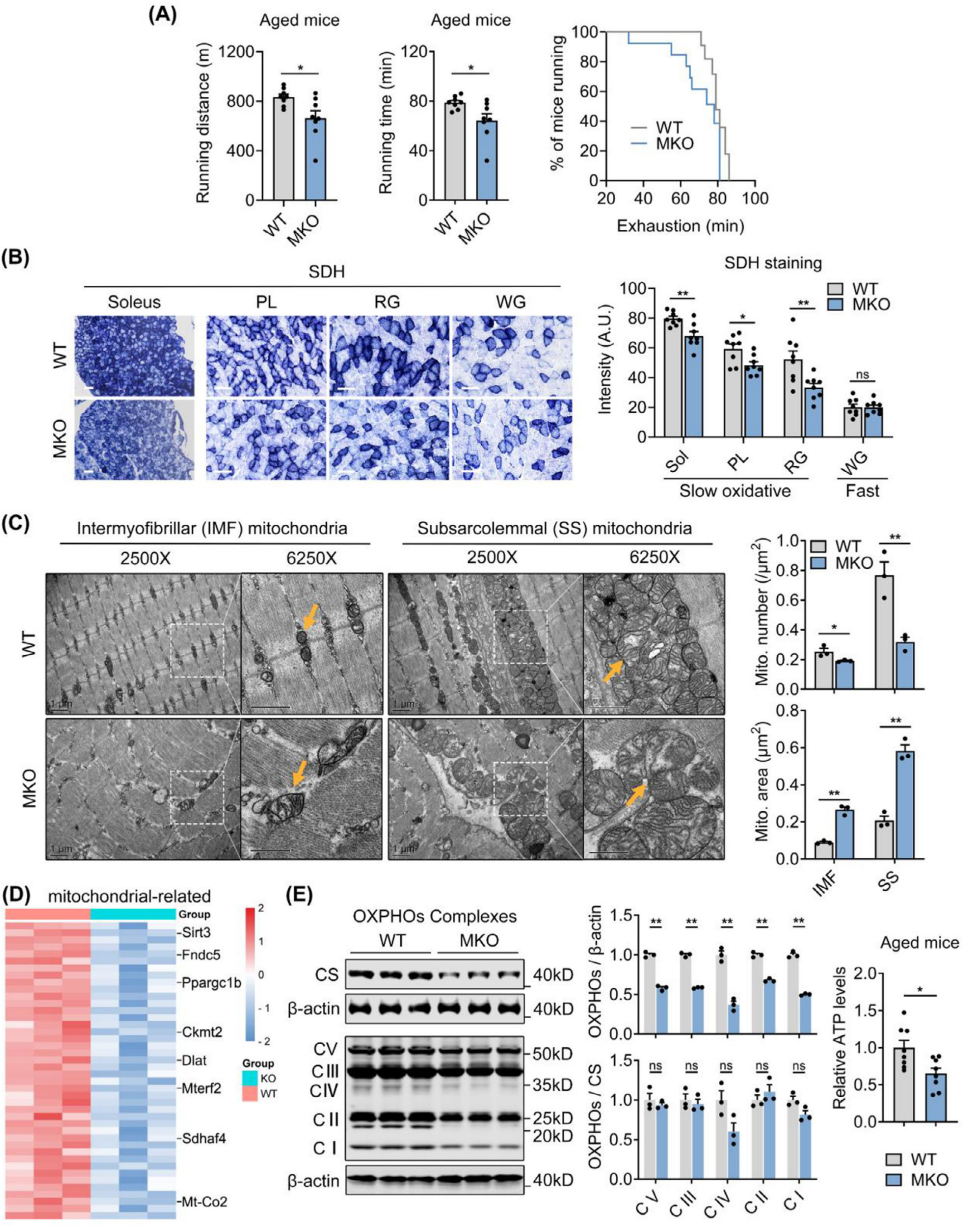
Muscle HSF1 deficiency deteriorates endurance capacity and causes mitochondrial dysfunction in oxidative fibers of aged mice. A) Treadmill endurance performance of indicated genotypes, including running distance (left), running time (middle), and running population plotted against time to exhaustion (right). B) Representative SDH staining in GAS/PL complex and soleus muscles of indicated genotypes (left) and quantification of SDH data (right). Note, the GAS/PL complex was further divided into three distinct portions based on fiber composition and metabolic profiles: plantaris (PL) and red gastrocnemius (RG), enriched with oxidative fibers, as well as white gastrocnemius (WG), characterized by a higher proportion of fast‐glycolytic fibers. Scale bar represents 100 µm. C) Representative electron micrographs of QU muscles showing intermyofibrillar (IMF) and subsarcolemmal (SS) mitochondria in sections of indicated genotypes and quantification of number and area of IMF and SS mitochondria (n = 3). Yellow arrows indicate mitochondria. Scale bar represents 1 µm. D) Heatmap analysis of mitochondrial‐related genes in GAS muscles of indicated genotypes. E) Representative western blot analysis of protein levels of OXPHOS complexes (n = 3; normalized to β‐actin and citrate synthetase) and ATP levels in GAS muscles of indicated genotypes. n = 8 mice per group unless otherwise noted. Statistical significance was assessed by unpaired Student's *t*‐test (A‐C and E). Data are presented as mean ± SEM and *p < 0.05; **p < 0.01; ns, not significant compared to control group.

Then, we performed a treadmill exhaustion test to further assess the muscle changes in aged HSF1‐MKO mice. Endurance performance, as shown by running time, running distance, and time to exhaustion, was markedly reduced in aged HSF1‐MKO mice (**Figure** **3**A), indicating that loss of HSF1 also led to impaired endurance capacity in muscles of aged mice. As endurance ability is tightly correlated with mitochondrial oxidation in muscle oxidative fibers, we performed succinate dehydrogenase (SDH) staining in aged HSF1‐MKO and WT mice, which is a marker for mitochondrial oxidative capacity. The gastrocnemius/plantaris (GAS/PL) muscle complex can be further divided into three distinct portions based on fiber composition and metabolic heterogeneity as the red gastrocnemius (RG) and plantaris (PL), enriched with oxidative fibers, and the white gastrocnemius (WG), characterized by a higher proportion of fast‐glycolytic fibers.^[^ ^34^, ^35^ ^]^ The results revealed significant decreases in SDH intensity of RG and PL muscle regions, as well as in slow oxidative fiber (Sol) muscle, but not in WG muscle regions (Figure 3B). Ultrastructural assessment of intermyofibrillar (IMF) and subsarcolemmal (SS) mitochondria in aged HSF1‐MKO muscles showed a significant reduction in mitochondria numbers, but the mitochondrial area was increased with a swelling morphology and disordered cristae structure, relative to WT mice (Figure 3C). Furthermore, transcriptome analysis revealed that mitochondrial function genes were markedly decreased in aged MKO muscles (Figure 3D), which was confirmed by decreased protein levels of mitochondrial OXPHOS complexes and ATP contents (Figure 3E). Besides, GSEA analysis results showed no enrichment for mitochondrial fission or fusion pathways in GAS from MKO versus WT mice (Figure S3F, Supporting Information), accompanied with no significant transcriptional changes in genes encoding mitofusins or Drp1 (Figure S3G, Supporting Information). Therefore, HSF1 deficiency in skeletal muscle may not affect mitochondrial fusion and fission pathways. Taken together, these data demonstrated that the absence of HSF1 in muscles of aged mice resulted in impaired mitochondrial functions in oxidative fibers that may partially compromise endurance capacity.

### 2.4 Muscle HSF1 Deficiency Aggravates Aging‐Associated Metabolic Dysfunction in Aged Mice

**Figure 4:**
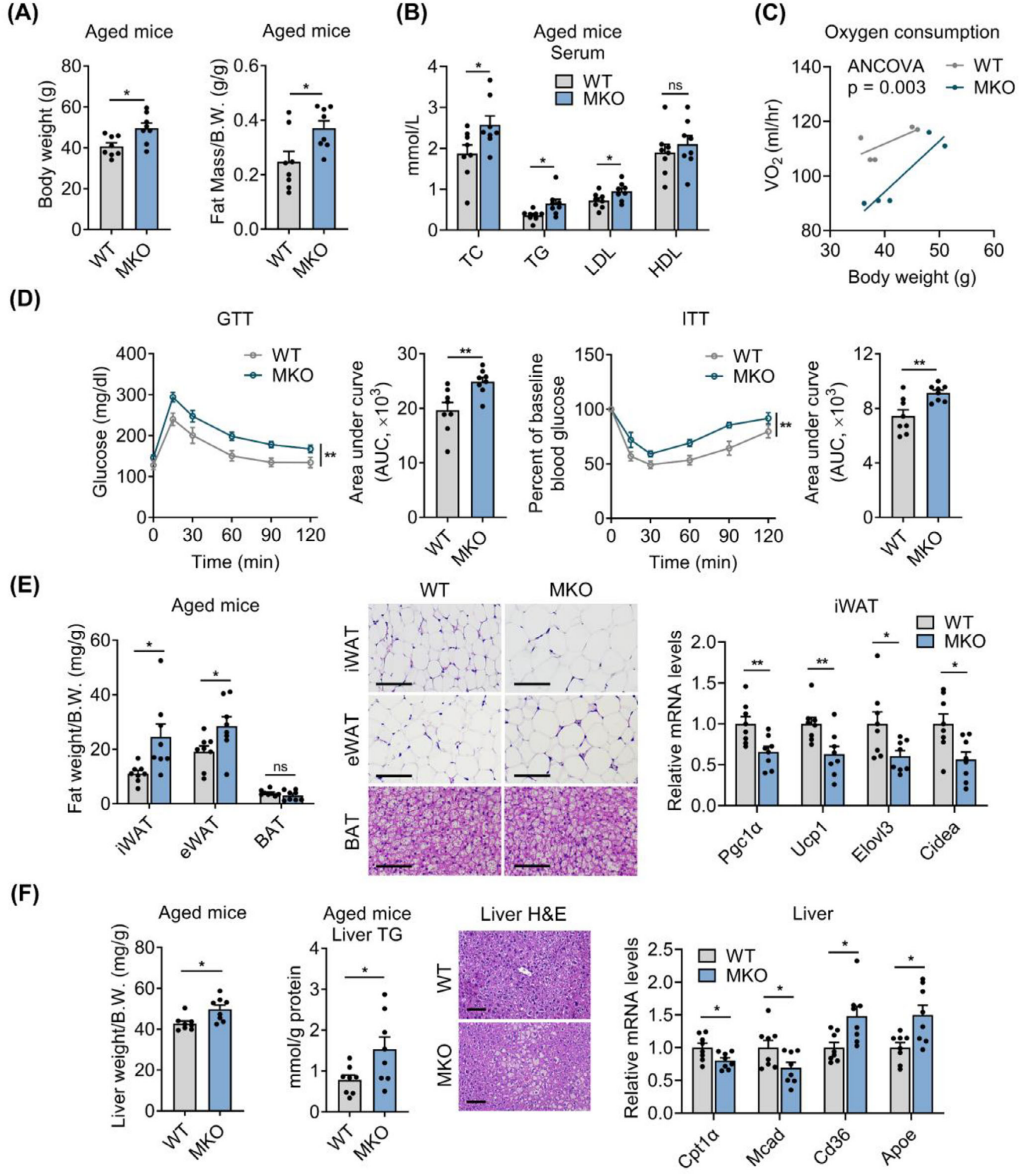
Muscle HSF1 deficiency aggravates aging‐associated metabolic dysfunction in aged mice. A–E) Analysis of metabolic parameters of aged HSF1‐MKO and WT mice, including (A) body weight (left), normalized fat mass (right); B) serum parameters, C) whole‐body oxygen consumption (right, n = 5); D) glucose tolerance test (GTT) and insulin tolerance test (ITT); E) adipose tissue weights (left), representative H&E staining of adipose tissues (middle), mRNA levels of thermogenic genes in iWAT (right); F) Liver weights and triglyceride (TG) (left), representative H&E staining of livers (middle) and mRNA levels of lipid transport and β‐oxidative genes in liver (right). Scale bar represents 100 µm. n = 8 mice per group unless otherwise noted. iWAT, inguinal white adipose tissue; eWAT, epididymal white adipose tissue; BAT, brown adipose tissue. Statistical significance was assessed by unpaired Student's t test (A, B, E, and F), ANCOVA with body weight as covariate (C), and two‐way ANOVA with Bonferroni's post hoc test (D). Data are presented as mean ± SEM and *p < 0.05; **p < 0.01; ns, not significant compared to control group.

Next, we evaluated the systemic metabolic changes in aged mice with HSF1 ablation in muscle. Our results unveiled that aged HSF1‐MKO mice exhibited significant increases in body weights mainly contributed by elevated fat mass, as well as serum lipidemia (**Figure** **4**A,B), accompanied with reduced oxygen consumption indicative of decreased energy expenditure, without alterations in food intake or locomotor activity (Figure 4C; Figure S3H, Supporting Information). Meanwhile, HSF1‐MKO mice showed impaired glucose tolerance and worsened insulin resistance (Figure 4D). In addition, we found that adipose tissue weights and average adipocyte sizes were significantly increased in aged HSF1‐MKO mice (Figure 4E), accompanied with decreased expression levels of thermogenic gene programs, including *Pgc1α*, *Ucp1*, *Elovl3*, and *Cidea* (Figure 4E). Besides, aged HSF1‐MKO mice showed increased liver weights, hepatic lipid accumulation and TG levels, possibly due to decreased levels of β‐oxidation genes and increased levels of lipid transport genes (Figure 4F). In summary, these results suggested that HSF1 deficiency in muscle exacerbated aging‐associated metabolic dysfunction in mice.

### 2.5 Active HSF1 Overexpression in Muscle Alleviates Aging‐Associated Muscle Atrophy and Mitochondrial Function Decline in Aged Mice

**Figure 5:**
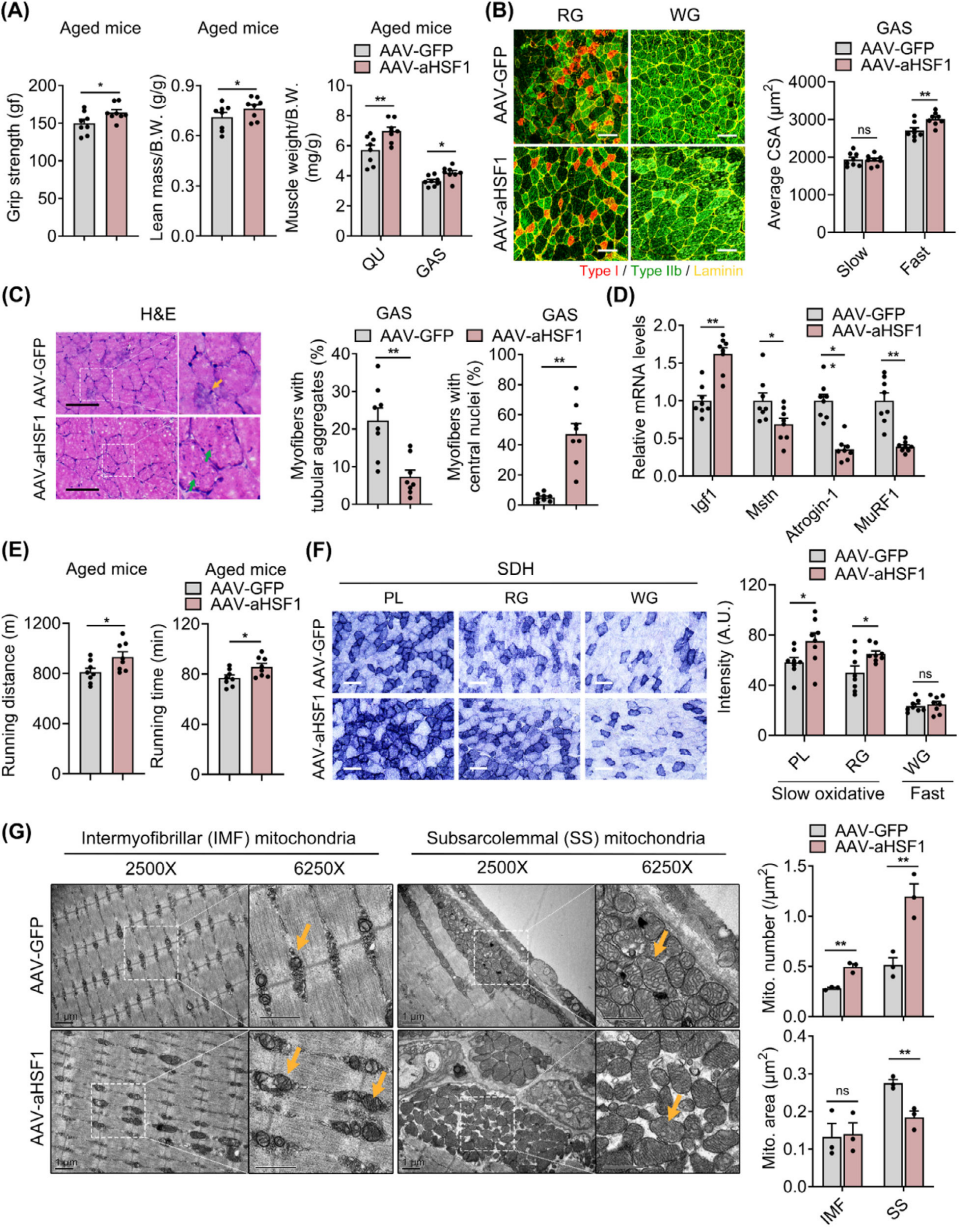
Active form of HSF1 overexpression in muscle alleviates aging‐associated muscle atrophy and mitochondrial function decline in aged mice. A) Whole limb grip strength (left), normalized lean mass (middle) and weights of QU and GAS muscles (right) from 22‐month‐old C57BL/6J male mice with intramuscular injection of AAV‐mediated GFP (AAV‐GFP) or active form of HSF1 overexpression (AAV‐aHSF1). B) Representative MyHC IF staining in GAS muscles of indicated genotypes (left) and quantification of fiber size of slow‐ and fast‐twitch fibers (right). Scale bar represents 100 µm. Red, type I myofibers; black, type IIa/x myofibers; green, type IIb myofibers; yellow, Laminin. C) Representative H&E staining (left), quantifications of myofibers with tubular aggregates (middle) and central nuclei (right) in GAS muscles of indicated groups. Yellow arrows indicate myofibers with tubular aggregates and green arrows indicate myofibers with central nuclei. Scale bar represents 100 µm. D) mRNA levels of atrophic genes in GAS muscles of indicated groups. E) Treadmill endurance performance of indicated groups, including running distance (left) and running time (right). F) Representative SDH staining and quantification of SDH intensity of indicated groups. Scale bar represents 100 µm. G) Representative electron micrographs of QU muscles showing IMF and SS mitochondria in sections of indicated groups and quantification of the number and area of IMF and SS mitochondria (n = 3). Yellow arrows indicate mitochondria. Scale bar represents 1 µm. n = 8 mice per group unless otherwise noted. Statistical significance was assessed by unpaired Student's *t*‐test (A‐G). Data are presented as mean ± SEM and *p < 0.05; **p < 0.01; ns, not significant compared to control group.

After evaluating the HSF1‐MKO caused defects in aged mice, we next studied if elevating HSF1 function in muscle would be protective during aging. We delivered AAV carrying active form of HSF1 (aHSF1), which is devoid of the LZ2 domain essential for repressing oligomerization and therefore remains continuously active, in the GAS/PL and QU muscle of aged mice and examined the phenotypes after 6 weeks (Figure S4A, Supporting Information).^[^ ^10^, ^21^ ^]^ Following intramuscular injection of AAV‐aHSF1, HSF1 expression levels were specifically and significantly elevated in GAS and QU muscles to comparable levels found in young muscle, with no ectopic expression in TA or other tissues (Figure S4B,C, Supporting Information). Besides, we further compared QU muscle morphology and gene changes in AAV‐GFP group with those in non‐injected mice as sham group. The results showed that AAV‐GFP caused no changes in QU muscle mass, size, atrophic and inflammatory gene expressions, compared to the muscle from non‐injected mice (Figure S4D–F, Supporting Information). AAV‐aHSF1 overexpression in GAS and QU muscles resulted in significant increases in grip strength, lean mass, and GAS and QU muscle weights compared to the control group (**Figure** **5**A), accompanied with comparable fiber‐type proportions and myofiber numbers, while larger fast‐twitch fiber sizes in mixed‐type muscle (Figure 5B; Figure S5A–C, Supporting Information), as well as lower occurrence of tubular aggregates (Figure 5C; Figure S5D, Supporting Information), indicating that HSF1 overexpression mitigated age‐associated fast‐twitch muscle degeneration. Interestingly, the percentage of myofibers with central nuclei was much higher in the AAV‐aHSF1 group (Figure 5C; Figure S5D, Supporting Information), suggesting that local muscular HSF1 elevation led to muscle regeneration, which was consistent with the previously reported role of HSF1 on muscle regeneration following CTX‐induced muscle injury.^[^ ^14^ ^]^ Molecular analysis revealed that the expression levels of MSTN, MAFbx and MuRF1 were downregulated, while IGF1‐mTOR‐S6K1 was increased in muscles with HSF1 active form overexpression (Figure 5D; Figure S5E, Supporting Information). Besides, consistent with HSF1 deficiency phenotype, HSF1 overexpression in muscle did not alter fibrosis (Figure S6A–C, Supporting Information). Overall, these data indicated that enhancing HSF1 activity in muscles alleviated aging‐induced muscle atrophy in mice mainly through increased fast‐twitch fiber sizes and muscle regeneration.

We further examined the physiological impact by assessing endurance performance in control or AAV‐aHSF1 aged mice. The results showed that the AAV‐aHSF1 group also showed increased running distance, running time and exhaustion time (Figure 5E; Figure S6D, Supporting Information), with consistently increased SDH staining intensity in oxidative PL and RG regions, but not in the glycolytic WG region (Figure 5F). Additionally, IMF and SS mitochondria numbers and integrity, as well as protein expression levels of mitochondrial OXPHOS complexes and ATP contents, were elevated in AAV‐aHSF1 treated group (Figure 5G; Figure S6E, Supporting Information).

Furthermore, we treated aged WT mice with celastrol, a HSF1 activator,^[^ ^12^ ^]^ and assessed the metabolic performance. Of note, celastrol diet ameliorated muscle sarcopenia in aged mice, including improved lean mass, muscle weights, fast glycolytic fiber sizes, running distance, mitochondrial function and SDH staining for slow oxidative fiber, accompanied with related gene programs (Figure S7, Supporting Information). These results suggested that celastrol mediated HSF1 activation may serve as a potent strategy against age‐associated sarcopenia.

Together, these results demonstrated that AAV‐mediated overexpression of active HSF1 in muscle and HSF1 pharmacological activation significantly ameliorated mitochondrial dysfunction and improved endurance performance in aged mice.

### 2.6 Muscle Active HSF1 Overexpression Ameliorates Aging‐Associated Metabolic Dysfunction

**Figure 6:**
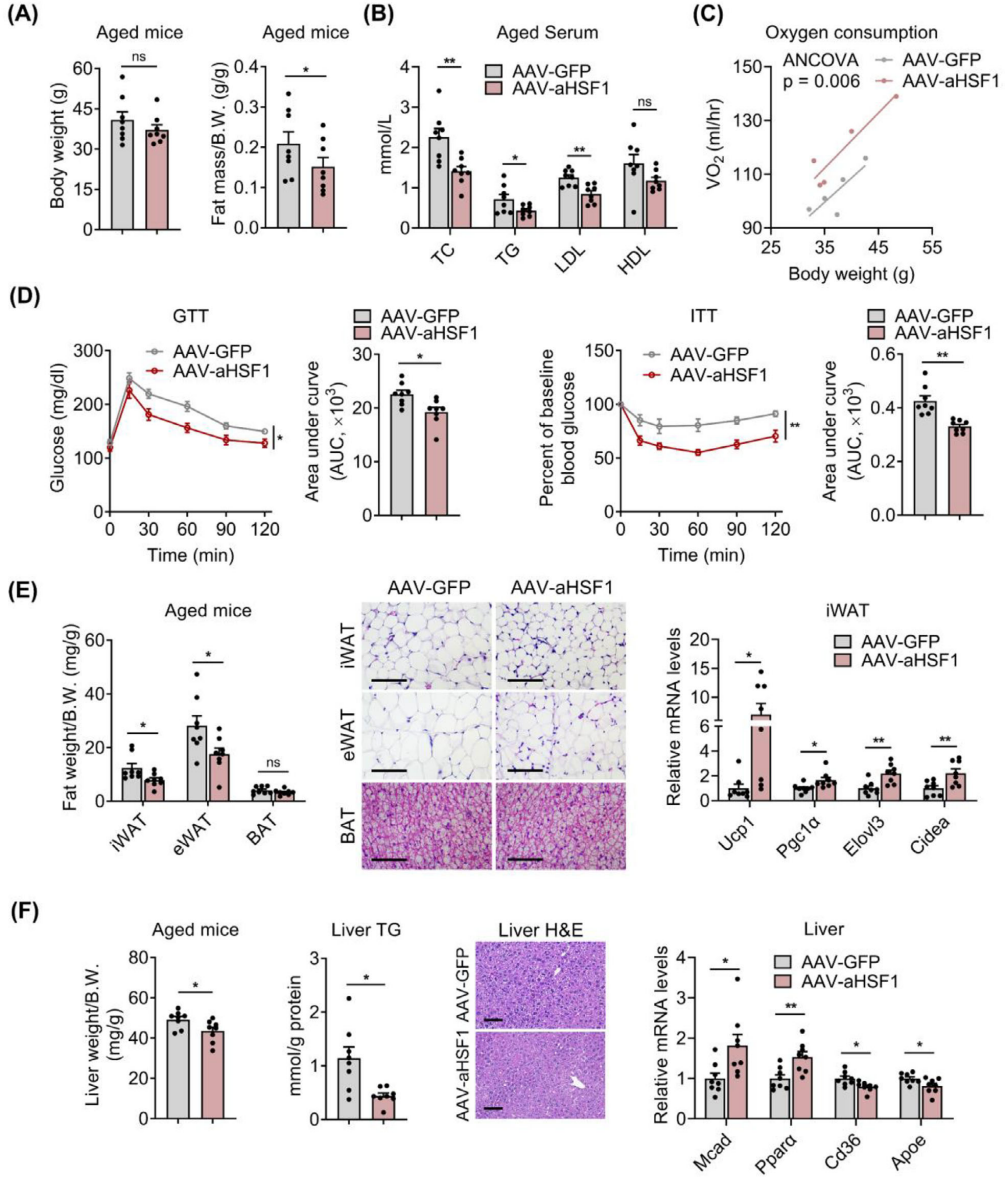
Muscle active HSF1 overexpression ameliorates aging‐associated metabolic dysfunction. A–F) Analysis of metabolic parameters of 22 months C57BL/6J male mice with intramuscular injection of AAV‐GFP or AAV‐aHSF1, including (A) body weight (left), normalized fat mass (right); B) serum parameters, C) whole‐body oxygen consumption (right, n = 5); D) GTT and ITT; E) adipose tissue weights (left), representative H&E staining of adipose tissues (middle), mRNA levels of thermogenic genes in iWAT (right); F) Liver weights and TG (left), representative H&E staining of livers (middle), and mRNA levels of lipid transport and β‐oxidative genes in liver (right). Scale bar represents 100 µm. n = 8 mice per group unless otherwise noted. iWAT, inguinal white adipose tissue; eWAT, epididymal white adipose tissue; BAT, brown adipose tissue. Statistical significance was assessed by unpaired Student's t test (A, B, E, and F), ANCOVA with body weight as covariate (C), and two‐way ANOVA with Bonferroni's post hoc test (D). Data are presented as mean ± SEM and *p < 0.05; **p < 0.01; ns, not significant compared to control group.

Furthermore, we found that the AAV‐aHSF1 treated group of aged mice exhibited reduced fat mass, improved lipid profiles, elevated energy expenditure, and enhanced glucose tolerance and insulin sensitivity, without changes in locomotor activity or food intake (**Figure** **6**A–D; Figure S8A, Supporting Information). Detailed morphological analysis revealed that the AAV‐aHSF1 group showed reduced adipose tissue weights, smaller adipocyte sizes and increased levels of browning genes in iWAT (Figure 6E). Besides, aging‐induced hepatic steatosis was improved, as evidenced by reduced liver weights and TG levels, accompanied with elevated expressions of genes for β‐oxidation and decreased genes for lipid uptake and transportation (Figure 6F). These data suggested that AAV‐mediated HSF1 overexpression in muscle ameliorated aging‐associated metabolic dysfunction.

### 2.7 HSF1 Regulates *Sirt3* Transcription and Mitigates Myotube Atrophy and Mitochondrial Dysfunction

**Figure 7:**
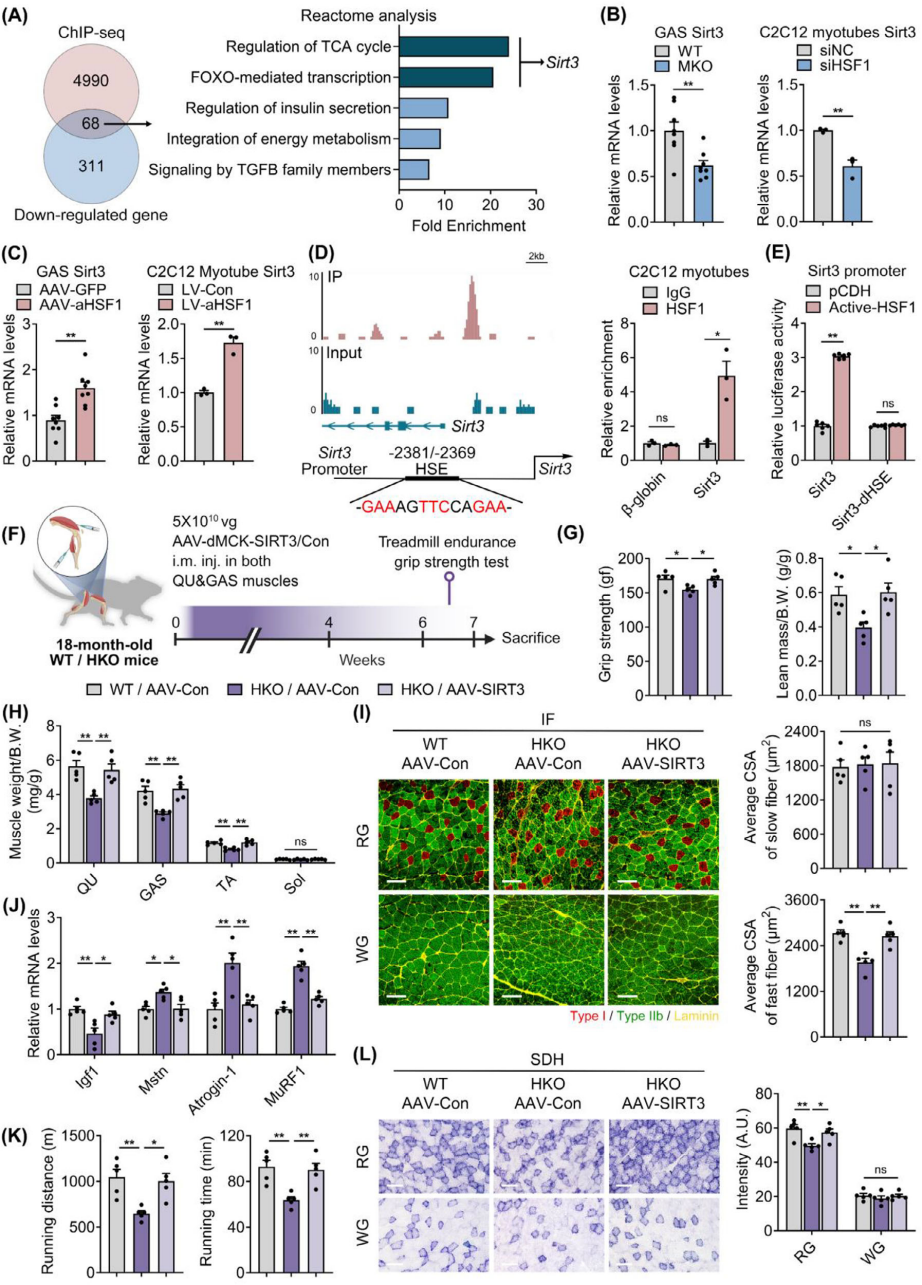
HSF1 regulates *Sirt3* transcription and mitigates muscle atrophy and mitochondrial dysfunction in vivo. A) Analysis of the HSF1 ChIP‐Seq data in C2C12 myotubes combined with RNA‐Seq dataset upon muscle HSF1 deletion defines a set of genes directly regulated by HSF1 (left) and Reactome pathway enrichment analysis of HSF1 direct targets, with the top 5 terms shown (right). B,C) mRNA level of *Sirt3* following HSF1 deficiency or activation. B) GAS muscles from WT or MKO mice (left, n = 8); C2C12 myotubes delivered with siNC or siHSF1 (right, n = 3); C) GAS muscles from AAV‐GFP or AAV‐aHSF1 group (left, n = 8); C2C12 myotubes transfected with Lentivirus‐Con (LV‐Con) or Lentivirus‐active HSF1 (LV‐aHSF1) (right, n = 3). D) HSF1 ChIP‐Seq tracks from myotubes at the *Sirt3* locus, one putative conserved HSE binding site within the promoter region of the *Sirt3* gene (left), and ChIP assay assessing HSF1 binding site in C2C12 myotubes (right, n = 3). E) Luciferase assay for validation of binding region (right, n = 6). F) Schematic illustration of AAV‐mediated SIRT3 overexpression in QU and GAS muscles of aged (18‐month‐old) mice. G) Grip strength (left), normalized lean mass (right), and H) muscle weights of indicated groups. I) Representative MyHC IF staining in GAS muscles (left) and quantification of fiber size of slow‐ and fast‐twitch fibers of indicated groups (right). Scale bar represents 100 µm. J) mRNA levels of atrophic genes in GAS muscles of the indicated groups. K) Treadmill endurance performance assessed as running distance (left) and time (right). L) Representative SDH staining and quantification of SDH intensity of indicated groups. Scale bar represents 100 µm. n = 5 mice per group unless otherwise noted. Statistical significance was assessed by unpaired Student's *t*‐test (B‐E) and one‐way ANOVA with Tukey's multiple comparisons (F and G). Data are presented as mean ± SEM and *p < 0.05; **p < 0.01; ns, not significant compared to control group.

To define the downstream target of HSF1 in the regulation of muscle physiology during aging, we performed HSF1 ChIP‐seq in myotubes and RNA‐seq in GAS of WT and HSF1‐MKO mice. Overlapping the two datasets rendered 68 genes, from which Reactome pathway analysis showed that the top terms were regulation of TCA cycle and FOXO‐mediated transcription, both closely associated with mitochondrial function and muscle atrophy. Notably, the top 2 terms share the only common DEG, sirtuin (Sirt) 3, which encodes a deacetylase enzyme important for mitochondrial function (**Figure** **7**A).^[^ ^36^ ^]^ We confirmed that in vivo and in vitro manipulations of HSF1 in muscle or myotubes led to changes in *Sirt3* transcription (Figures 3D and 7B,C). In addition, ChIP‐seq revealed a distinct enrichment of HSF1 binding on a canonical heat shock element (HSE) on the *Sirt3* promoter region (Figure 7D), which was confirmed by ChIP assay (Figure 7D). Besides, luciferase assay showed that overexpression of active form HSF1 significantly induced *Sirt3* promoter transcriptional activation, while the effects were fully blunted by deletion of the HSE region on *Sirt3* promoter (Figure 7E).

We then performed in vivo studies to assess the HSF1‐SIRT3 axis in mice muscles. We used aged HSA‐Cre HSF1‐loxp mice model as muscle‐specific HSF1 knockout mice (HSF1‐HKO) to avoid possible heart leaky (Figure S9A–C, Supporting Information), and overexpressed SIRT3 in GAS and QU muscles via dMCK promoter driven AAV (AAV‐MCK‐SIRT3). Besides, we also achieved SIRT3 pharmacological activation via daily intraperitoneal injections of HKL (5 mg kg^−1^ body weight), a SIRT3 activator, for 2 weeks. Of note, intramuscular injection of AAV‐SIRT3 restored *Sirt3* expression in muscles (Figure 7F; Figure S9D, Supporting Information), thus ameliorating muscle performance, age‐associated muscle atrophy and mitochondrial dysfunction in HSF1 muscle deficient mice, including reduced grip strength, lean mass/body weights (Figure 7G), muscle weights/body weights (Figure 7H), fast glycolytic fiber sizes, accompanied with related gene programs (Figure 7I,J), running distance, running time (Figure 7K; Figure S9E, Supporting Information), SDH staining of slow oxidative fibers, ATP levels in GAS muscles and oxygen consumption rate (OCR) in C2C12 myotubes (Figure 7L; Figure S9F–H, Supporting Information). In addition, pharmacological activation of SIRT3 in muscle significantly alleviated grip strength, tetanic contraction force of extensor digitorum longus (EDL, fast‐twitch muscle), lean mass/body weights and muscle weights/body weights (Figure S10A–D, Supporting Information). Besides, the rescue group showed larger fast‐twitch fiber sizes and comparable slow‐twitch fiber sizes in GAS muscles, accompanied with atrophic gene programs (Figure S10E,F, Supporting Information). Furthermore, treadmill endurance performance, as shown by running distance and time, was improved in the SIRT3 rescue group, with consistently increased SDH staining intensity in oxidative RG regions, but not in the glycolytic WG region (Figure S10G,H, Supporting Information).

Furthermore, we found that HSF1 knockdown in DEX‐treated myotubes, a classic atrophic model, significantly deteriorated myotube atrophy, as evidenced by decreased myotube diameters and increased muscle atrophic gene expression. Of note, the effects of HSF1 deficiency could be rescued by administration of HKL (Figure S11A, Supporting Information). Meanwhile, HSF1 knockdown in normal myotubes caused mitochondrial function decline, as indicated by reduced mitochondrial numbers with TOM20 staining, lower ATP contents, and downregulation of mitochondrial genes, while HKL treatment blunted these effects (Figure S11B, Supporting Information). Consistently, HSF1 overexpression in DEX‐treated myotubes increased myotube diameter and decreased atrophic gene expression, while HSF1 overexpression in normal myotubes increased mitochondrial numbers, ATP production and mitochondrial gene expression, which could be abrogated by SIRT3 inhibitor 3‐TYP treatment (Figure S11A,B, Supporting Information). These in vivo and in vitro data suggested that SIRT3 might be a direct downstream target gene of HSF1 for the regulation of both muscle atrophy and mitochondrial functions.

### 2.8 SIRT3 Deacetylates both PGC1α1 and PGC1α4 to Exert Beneficial Effects on Muscle and Induces Crosstalk Between Muscle and Adipose Tissues via Irisin

**Figure 8:**
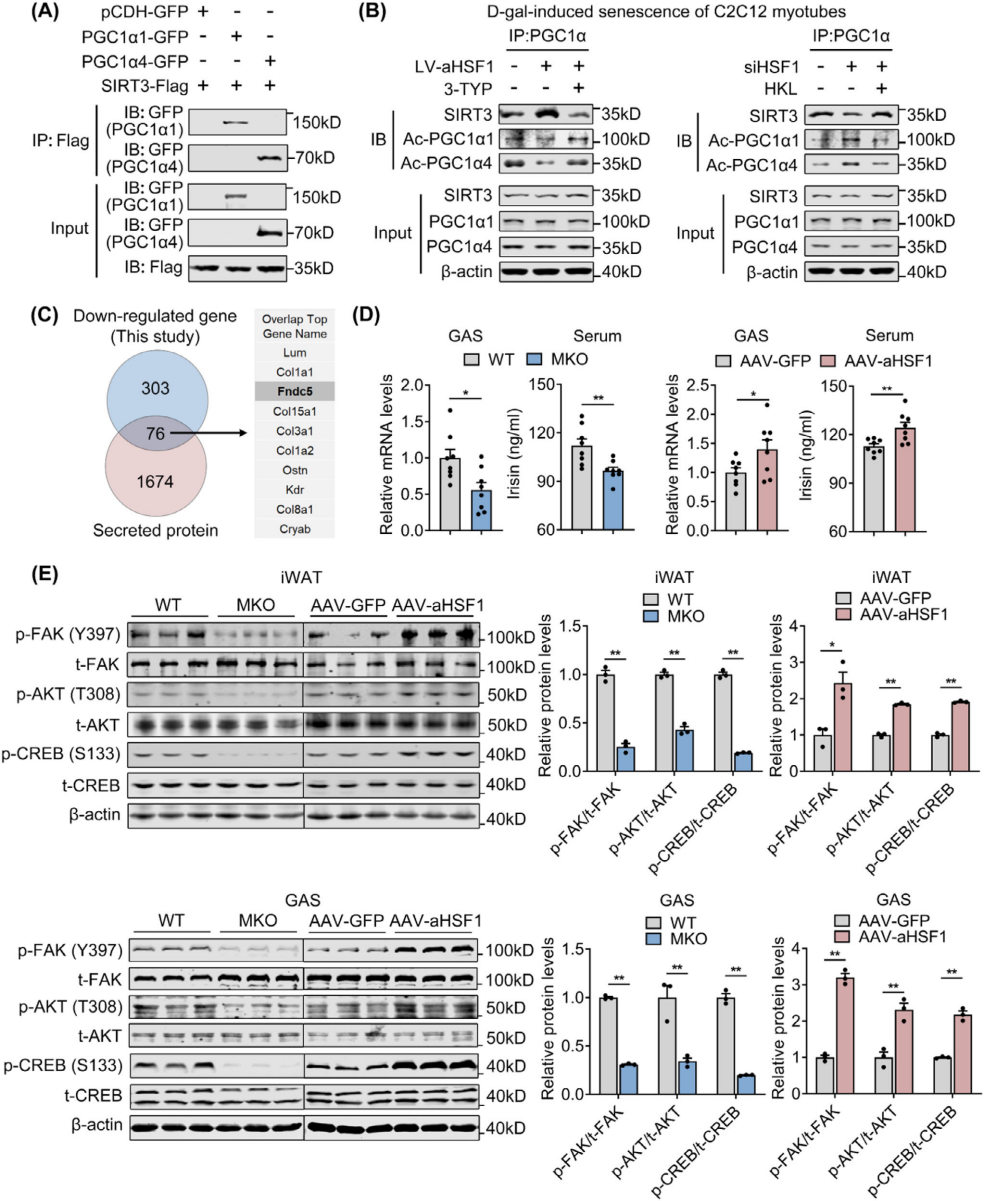
SIRT3 deacetylates both PGC1α1 and PGC1α4 to exert beneficial effects on muscle and induces possible crosstalk between muscle and adipose tissues via Irisin. A) Co‐immunoprecipitation (Co‐IP) experiments were performed by co‐transfecting PGC1α1‐GFP/PGC1α4‐GFP and SIRT3‐Flag in HEK293T cells, with detailed treatment information indicated in the panel above. Antibodies against the Flag epitope were used for co‐IP. Extracts (Input) from the HEK293T cells and proteins from the IP were analyzed by immunoblotting. Representative results for co‐IP are shown. n = 3 independent experiments. B) Representative immunoblots of endogenous co‐IP analysis showing SIRT3 and PGC1α acetyl‐lysine (Ac‐Lys) levels in the IP, as well as levels of the indicated proteins in the input. n = 3 independent experiments. C) Analysis of RNA‐seq dataset upon muscle HSF1 deletion combined with a set of mouse secreted proteins defines a list of secreted proteins regulated by HSF1, ranked by adjusted P‐value. D) mRNA and serum levels of FNDC5/Irisin following HSF1 deficiency or activation. n = 8 mice per group. E) Representative western blot analysis and quantification of integrin‐like signaling FAK‐AKT‐CREB phosphorylation in iWAT and GAS from MKO or AAV‐aHSF1 mice and their controls. n = 3 per group. Statistical significance was assessed by unpaired Student's *t*‐test (D and E). Data are presented as mean ± SEM and *p < 0.05, **p < 0.01 compared to control group.

PGC1α plays a complex regulatory role in metabolic processes and cellular fate determination. PGC1α1 and PGC1α4 are two major isoforms of PGC1α, with PGC1α1 promoting mitochondrial biogenesis in muscle, while PGC1α4 boosting muscle hypertrophy.^[^ ^18^ ^]^ The activity of PGC1α is robustly modulated transcriptionally and post‐translationally, notably through acetylation/deacetylation, which affects both its activity and nuclear localization.^[^ ^37^ ^]^ SIRT3 is a mitochondrial deacetylase, though evidences suggest SIRT3 may exert functions outside of mitochondria.^[^ ^38^ ^]^ For example, SIRT3 enhances PGC1α’s coactivation of nuclear‐encoded mitochondrial genes.^[^ ^39^ ^]^ Furthermore, SIRT3 antagonizes stem cell senescence in the nucleus via epigenetic mechanisms.^[^ ^40^ ^]^ Given these findings, we hypothesized that SIRT3 might physically interact with both PGC1α1 and PGC1α4 in nucleus and deacetylate these proteins to alleviate muscle atrophy and mitochondrial dysfunction. Indeed, we performed co‐IP assays and found that both PGC1α1 and PGC1α4 could interact with SIRT3 (**Figure** **8**A). In addition, endogenous co‐IP experiments in D‐gal‐induced senescent C2C12 myotubes demonstrated that HSF1 overexpression enhanced the interaction between SIRT3 and both PGC1α isoforms, resulting in reduced PGC1α acetylation of both isoforms (Figure 8B; Figure S12A, Supporting Information). Conversely, HSF1 knockdown suppressed the interaction and increased PGC1α acetylation of both isoforms. Notably, the SIRT3 inhibitor 3‐TPY and activator HKL could abolish the effects induced by HSF1 manipulation (Figure 8B; Figure S12A, Supporting Information). In addition, we performed endogenous co‐IP assay and validated the interaction of PGC1α isoforms with SIRT3 in GAS muscle from aged WT/AAV‐Con, HSF1‐HKO/AAV‐Con and HSF1‐HKO/AAV‐SIRT3 groups of mice. The results demonstrated HSF1 deficiency reduced interaction of PGC1α and SIRT3 and increased acetylated PGC1α1 and PGC1α4, which were rescued by SIRT3 overexpression in muscle (Figure S12B,C, Supporting Information). Overall, these data suggested that PGC1α interacted with SIRT3 both in vitro and in vivo.

Furthermore, we integrated the down‐regulated genes from RNA‐seq of the muscle of HSF1‐MKO mice with the Secreted Protein Database (SEPDB) and found that fibronectin type III domain‐containing protein 5 (FNDC5)/Irisin, a previously reported PGC1α downstream key peptide for muscle functionality and fat biology, was altered (Figure 8C).^[^ ^41^, ^42^ ^]^ Indeed, we confirmed that *Fndc5* mRNA levels in GAS muscle and Irisin levels in serum were reduced in aged HSF1‐MKO mice, while elevated in the AAV‐aHSF1 group of aged mice (Figure 8D). Besides, Irisin signaling of FAK‐AKT‐CREB in adipose tissues and muscles was altered consistently (Figure 8E), suggesting irisin signaling activation. This is also consistent with the improved adipose tissue function in GAS aHSF1‐overexpression mice. To functionally link the systemic phenotypes to Irisin, we further established in vitro muscle‐adipose/liver crosstalk models (Figure S13, Supporting Information). In the muscle‐adipose model, conditioned medium (CM) from HSF1‐knockdown myotubes suppressed beige adipocyte browning (Figure S13C, Supporting Information), while CM from HSF1‐overexpressing myotubes enhanced this process (Figure S13D, Supporting Information). These effects were dependent on Irisin, as supplementation with recombinant Irisin restored browning capacity (Figure S13C, Supporting Information), whereas an Irisin neutralizing antibody abolished the enhanced browning induced by HSF1‐overexpression CM (Figure S13D, Supporting Information). Similarly, in the muscle‐liver model, CM from HSF1‐knockdown myotubes promoted hepatic lipid accumulation (Figure S13F, Supporting Information), while CM from HSF1‐overexpressing myotubes attenuated steatosis (Figure S13G, Supporting Information). Again, these effects were Irisin‐dependent, as recombinant Irisin ameliorated lipid accumulation (Figure S13F, Supporting Information), and Irisin neutralization blunted the protective effects of HSF1‐overexpression CM (Figure S13G, Supporting Information). Collectively, these data suggested that Irisin is indispensable for HSF1 regulated crosstalk to metabolic organs, including adipose tissue and livers, thus establishing a functional link between the circulating Irisin changes and systemic metabolic performance. Together, our results suggested that muscle HSF1 enhanced the transcription of *Sirt3*, which interacted with and deacetylated both PGC1α1 and PGC1α4 to prevent myotube atrophy and promote mitochondrial function, as well as improve systemic metabolic performances in aged mice, possibly via Irisin.

## 3 Discussion

**Figure 9:**
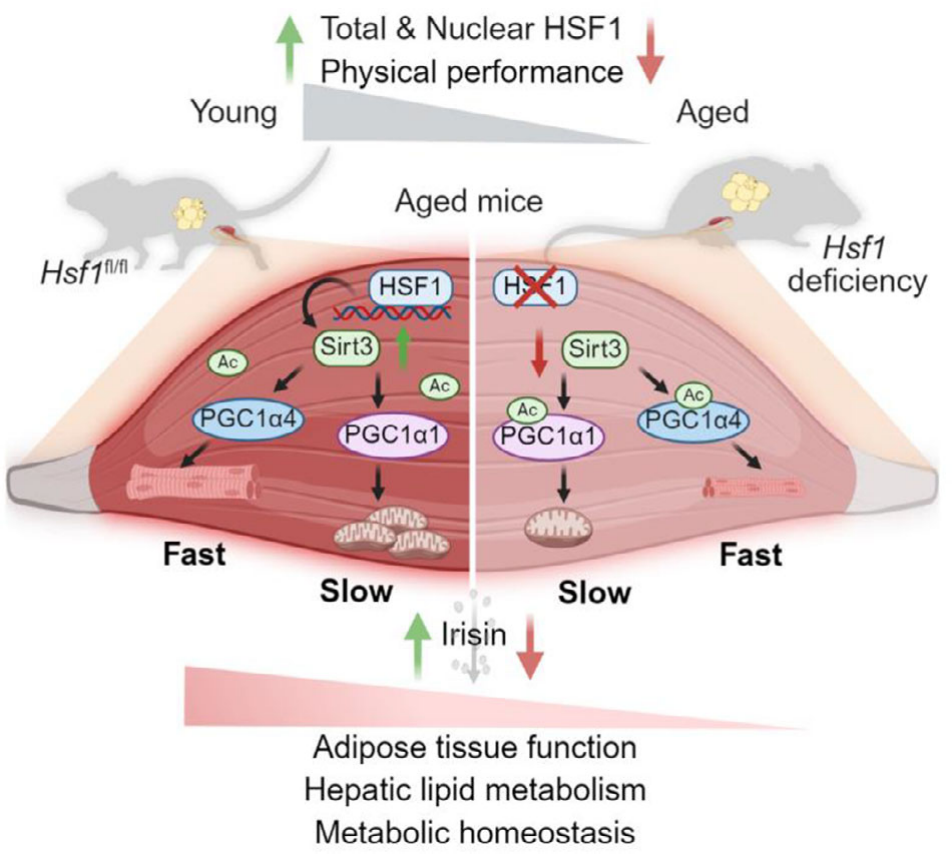
Schematic Illustration of the role of skeletal muscle HSF1 in alleviation of age‐associated sarcopenia and mitochondrial function decline via SIRT3‐PGC1α axis. Summary of the HSF1‐SIRT3‐PGC1α axis in muscle aging. HSF1 decline with age disrupts SIRT3‐mediated deacetylation of PGC1α isoforms, driving fiber atrophy and mitochondrial dysfunction in a fiber‐type‐specific manner. Therapeutic activation of this pathway improves muscle health and stimulates Irisin secretion, which possibly mediates muscle‐fat/liver cross‐talk.

Heat shock factor 1 (HSF1) is a master regulator of the heat shock response, orchestrating the transcription of *Hsps* responsible for proteotoxic stress protection and cellular homeostasis.^[^ ^9^ ^]^ *Hsf1* has been considered a pro‐longevity gene since HSF1 deficiency reduced lifespan and health span in *Caenorhabditis* *elegans* and mice under stress conditions.^[^ ^43^, ^44^ ^]^ Besides, we and others have found that *Hsf1* transcription levels declined in multiple tissues, including muscle, heart and brain, during aging, suggesting the potential of targeting HSF1 for aging‐related diseases.^[^ ^45^, ^46^ ^]^ In this study, we found that muscle HSF1 deficiency in aged mice led to deteriorated age‐related sarcopenia and metabolic dysfunction, while AAV‐mediated muscle overexpression of active HSF1 could ameliorate these defects by enhancing mitochondrial function and muscle performance. Considering that we recently reported HSF1 activation in adipose tissues via local hyperthermia prevented and treated obesity,^[^ ^10^ ^]^ these data suggested HSF1 as a potential therapeutic target for obesity and age‐associated metabolic dysfunctions (**Figure** **9**).

Canonical HSF1 target genes, such as HSP70, have been shown to be beneficial for combating dexamethasone‐induced muscle atrophy.^[^ ^47^ ^]^ Specifically, HSP70 may activate the anabolic Akt1 and ERK1/2 pathways and suppress the catabolic FoxO3‐MuRF1‐proteasome pathway.^[^ ^48^ ^]^ Besides, HSF1 has been shown to activate ATF3 to inhibit the expression of IL‐6, which is the key inflammatory factor that impairs muscle fiber structure and enhances protein breakdown.^[^ ^49^, ^50^ ^]^ Of note, hyperthermia therapy (HT) has long been recognized as an efficient avenue to activate HSF1 and convey various health effects, particularly in musculoskeletal injuries, by increasing the temperature of the skin and muscles, thereby stimulating local blood flow and metabolism.^[^ ^51^ ^]^ Besides, HT ameliorates eccentric exercise‐induced muscle injury and immobilization‐ or synergist ablation‐induced muscle atrophy, and improves muscle regeneration by increased HSPs and activation of mTOR signaling or sarcoplasmic/endoplasmic reticulum Ca^2+^‐ATPase (SERCA) activity.^[^ ^51^ ^]^ HT also promotes mitochondrial biogenesis through the activation of the AMPK‐SIRT1‐PGC1α pathway and reduces inflammation and oxidative damage,^[^ ^51^, ^52^ ^]^ overall suggesting the potential application of using HT against age‐associated sarcopenia. It would thus be promising to study the effects of HT on aging‐associated sarcopenia.

Via HSF1 ChIP‐seq, we have identified *Sirt3* as a key HSF1 target gene in muscle. SIRT3 is a crucial sirtuin within mitochondria. It is also a pro‐longevity gene that plays a pivotal role in maintaining mitochondrial homeostasis.^[^ ^39^ ^]^ The deletion of *Sirt3* results in impaired glucose oxidation in muscles due to mitochondrial protein hyperacetylation and decreased pyruvate dehydrogenase and hexokinase 2 activities,^[^ ^53^, ^54^ ^]^ highlighting the importance of SIRT3 in maintaining muscle metabolic flexibility. Besides, SIRT3‐mediated antioxidant defense inhibits skeletal muscle atrophy.^[^ ^55^ ^]^ Given that levels of human SIRT3 protein in serum and muscle tissues also decrease with aging, HSF1 and SIRT3 appear to function synergistically,^[^ ^56^, ^57^ ^]^ suggesting potential applications of targeting the HSF1‐SIRT3 axis against aging. Interestingly, Fernandez‐Marcos et al. found minimal basal phenotype in muscle‐specific SIRT3‐KO mice under both chow and HFD conditions.^[^ ^58^ ^]^ It has to be noted that both chow and HFD conditions pose few metabolic challenges for muscle sarcopenia. Meanwhile, we have studied the SIRT3 function in the aging scenario, suggesting the role of SIRT3 may be manifested under specific stresses, such as aging, which was in accordance with previous reports showing that SIRT3 deficiency may also deteriorate cardiac dysfunction in aged mice.^[^ ^59^, ^60^ ^]^ SIRT3 exists in two primary isoforms (long/M1 or M2 and short/M3) expressed in both humans and mice.^[^ ^61^, ^62^ ^]^ The long isoform, while detectable both in the cytoplasm and nucleus, predominantly resides in the mitochondria.^[^ ^38^, ^61^, ^62^ ^]^ In contrast, the M3 splice variant lacks the mitochondrial targeting sequence, resulting in its localization only in the cytoplasm; it also exhibits poor basal stability and is prone to ubiquitination and rapid degradation.^[^ ^63^, ^64^ ^]^ The possible paradoxical finding of Lin et al., where they studied the short M3 splice variant and found that short SIRT3 isoform overexpression reduced muscle mass.^[^ ^65^ ^]^ In the present study, the function of the full *Sirt3* variant was analyzed and AAV‐mediated rescue was also performed with the full‐length *Sirt3* transcript, which may explain the differential effects we observed.

We have analyzed HSF1 target genes in young HSF1‐MKO mice and found no change of these genes (Figure S14A, Supporting Information). In young organisms, cellular homeostasis is well balanced with multiple compensatory pathways that may resist to HSF1 deficiency caused defects in muscle gene programs. For example, the IRE1α pathway of the unfolded protein response regulates skeletal muscle size by promoting regeneration and hypertrophy.^[^ ^66^ ^]^ E2‐related factor 2 pathway improves mitochondrial function by activating antioxidant enzymes to reduce ROS levels, thereby protecting the respiratory chain, membrane permeability, and calcium homeostasis.^[^ ^67^ ^]^ In addition, activated AMPK can phosphorylate and activate PGC1α, promoting mitochondrial biogenesis and energy metabolism.^[^ ^68^ ^]^ However, during aging, the decline of these compensatory mechanisms may render the system highly dependent on the HSF1‐SIRT3 axis, thus contributing to the age‐dependent change in HSF1 target genes.

PGC1α has been shown to protect against sarcopenia, age‐related metabolic disorders such as type 2 diabetes mellitus and hepatic steatosis, as well as immobilization‐induced muscle atrophy.^[^ ^69^, ^70^, ^71^, ^72^ ^]^ PGC1α1 and PGC1α4 are two major isoforms of PGC1α that play distinct roles in mitochondrial functions and muscle hypertrophy.^[^ ^21^, ^72^ ^]^ HSF1 has been shown to directly activate *Pgc1α* transcription in skeletal muscle.^[^ ^12^ ^]^ In the present study, we deciphered the differential impact of HSF1 on muscle hypertrophy and oxidative capacity. Via MyHC IF, we found that fast fibers are more sensitive to fiber size changes upon HSF1 fluctuation, thus influencing muscle hypertrophy, while HSF1 impacted mitochondrial physiology mainly in oxidative fibers by assessing the changes in fiber oxidative functions via SDH staining and TEM analysis. The distinct functional impacts of HSF1 on fast and slow fibers in aging may due to the fiber type‐specific intrinsic gene programs. We found that HSF1 expression was similar between young tibialis anterior (TA, fast‐twitch) and soleus (Sol, slow‐twitch) muscles, which were both decreased in aged mice at similar magnitude (Figure S14B, Supporting Information). However, *Pgc1α1*, the master isoform regulating mitochondria biogenesis, was specifically decreased in aged Sol (Figure S14C, Supporting Information), while *Pgc1α4*, the master isoform regulating hypertrophy, was specifically decreased in aged TA (Figure S14D, Supporting Information). During aging, HSF1 may induce SIRT3 for PGC1α1 and PGC1α4 deacetylation and functional enhancement to counteract their expression decreases in muscles. HSF1 knockout ablated this crucial support, in turn, decreased *Pgc1α1* and *Pgc1α4* levels in slow and fast fibers, respectively, causing fiber‐specific phenotypes. Thus, the specific expression patterns of *Pgc1α1* and *Pgc1α4* on specific fiber types in aging may explain the overall contribution of HSF1 in the regulation of muscle function of aged mice.

Furthermore, Irisin, a myokine from the proteolytic cleavage of FNDC5 protein, has been shown to be induced by endurance training through PGC1α1 and by resistance training through PGC1α4.^[^ ^42^, ^73^ ^]^ It is possible that irisin mediated crosstalk between different muscle groups and adipose tissues, which functioned via an autocrine or paracrine fashion on muscles and adipose tissues to induce muscle hypertrophy and maintain systemic metabolic health.^[^ ^42^, ^73^ ^]^ Thus, spatial transcriptome, proteomics and metabolomics would be helpful to further understand muscle physiology in different fiber types under the HSF1‐SIRT3‐PGC1α‐Fndc5/Irisin regulatory cascade.

## 4 Conclusion

In summary, we demonstrated a specific and critical role of HSF1 in skeletal muscle via its regulation of SIRT3‐PGC1α axis, which impacts mitochondrial function in slow myofibers and muscle hypertrophy in fast myofibers via PGC1α1 and PGC1α4, respectively, therefore highlighting the comprehensive role of HSF1 in muscle biology and systemic energy homeostasis (Figure 9).

## 5 Experimental Section

### Human Muscle Sample Collection and Analysis

To evaluate the expression levels of HSF1 in human muscle samples, vastus lateralis muscle biopsies were obtained from a diverse group of male patients aged 13–88 years. These individuals were selected from those receiving orthopedic surgery at Shanghai Jiao Tong University Affiliated Sixth People's Hospital. Following collection, the biopsies were conducted to qPCR and subsequent statistical analysis. The human study was approved by the Human Research Ethics Committee of Shanghai Jiao Tong University Affiliated Sixth People's Hospital (2022‐YS‐095). Written informed consent was obtained from all subjects. Pearson correlation analysis of the mRNA levels of *HSF1*, *MSTN*, and *TFAM* genes in human muscle tissues was performed using RNA‐seq data from the Genotype‐Tissue Expression (GTEx) v10 database.

### Animal Studies

All animal experiments were performed in accordance with procedures by the Animal Ethics Committee of East China Normal University (ARXM2023019). Mice were maintained under specific‐pathogen‐free (SPF) conditions with a standardized 12‐h light/12‐h dark cycle and had free access to sterile water and food. The study involved C57BL/6J male mice at the ages of 3 months and 22 months to compare the physiological responses of young and aged individuals. Muscle‐specific HSF1 knockout mice were generated by crossing *Hsf1* ^loxp/loxp^ mice (kindly provided by Dr. Richard L.Prioa and Dr. Elisabetta Mueller from NIDDK, NIH) with transgenic mice expressing Cre recombinase under the control of the muscle‐specific muscle creatine kinase (MCK) promoter (Jax Lab, 006475) or the human skeletal actin (HSA) promoter (Jax Lab, 006139). *Hsf1* ^loxp/loxp^;*MCK‐Cre* (herein named HSF1‐MKO) mice and wild‐type (WT) littermates were analyzed at 3, 12, and 22 months of age. In rescue experiments in vivo, *Hsf1* ^loxp/loxp^;*HSA‐Cre* (HSF1‐HKO) mice and their littermates were examined at 18 months. Body composition was measured by the AccuFat‐1050 NMR system (MAGMED) and body weight was monitored weekly. For assessing metabolic parameters, mice were housed individually in Comprehensive Lab Animal Monitoring System (CLAMS, Columbus Instruments) cages. Oxygen consumption, carbon dioxide production, and energy expenditure were recorded.

### Cell Culture and Treatments

C2C12 murine myoblasts (CRL‐1772) and HEK293T cells (CRL‐3216) were obtained from the American Type Culture Collection (ATCC) and had been confirmed to be mycoplasma‐free by PCR testing. The cells were cultured in the growth medium (GM) made of Dulbecco's modified Eagle's medium (DMEM) (C11995500BT, Gibco) with 10% fetal bovine serum (A5256701, Gibco) and 1% penicillin and streptomycin (P/S) (15070063, Gibco) at 37 °C in a 5% CO_2_ and 95% air‐humidified atmosphere. For C2C12 differentiation, C2C12 myoblasts were plated in a 6 or 12‐well plate. When cells grew up to 80–90% confluency, the medium was replaced with differentiation medium (DM) containing DMEM supplemented with 2% horse serum (SH30074.03, HyClone), 1 µM insulin (I9278, Sigma–Aldrich), and 1% P/S. After 5 days of treatment with DM, the C2C12 myoblasts were differentiated into myotubes. To induce myotube atrophy in vitro, differentiated myotubes were treated with dexamethasone (DEX, 50 µM, HY‐14648, MedChemExpress) or vehicle for 24 h and then subjected to rescue experiments.^[^ ^74^ ^]^ To induce cellular senescence, differentiated myotubes were treated with D‐galactose (D‐gal, 30 g L^−1^, G0750, Sigma) or vehicle for 24 h.

To investigate the effects of HSF1 overexpression and SIRT3 inhibition on C2C12 myotubes, cells were transduced with the indicated lentivirus (LV‐Con or LV‐active HSF1) for 24 h, followed by treatment with the SIRT3 inhibitor 3‐TYP (50 µM, HY‐108331, MedChemExpress) dissolved in DMSO or vehicle for an additional 24 h.^[^ ^75^ ^]^ To determine the effects of HSF1 knockdown and SIRT3 activation on C2C12 myotubes, cells were transfected with siNC or siHSF1 (GenePharma, China) using Lipofectamine RNAiMAX (13778075, Thermo Fisher) for 24 h according to the manufacturer's protocol. The siRNA sequences are provided in Table S1 (Supporting Information). After infection, cells were incubated with the SIRT3 activator Honokiol (HKL, 20 µM, HY‐N0003, MedChemExpress) dissolved in DMSO or vehicle for an additional 24 h.^[^ ^76^ ^]^ Cells were harvested for in vitro molecular or morphological analysis after incubation.

To examine the paracrine effects of C2C12‐derived conditioned medium on adipocytes and hepatocytes, immortalized beige preadipocyte line and AML12 (CRL‐2254, ATCC) cells were used. The immortalized beige preadipocytes line was maintained and differentiated into beige adipocytes as previously described.^[^ ^10^ ^]^ AML12 hepatocytes were cultured in DMEM/F12 (11330032, Invitrogen) supplemented with 10% FBS, 1% insulin‐transferrin‐selenium liquid media supplement (I3146, Sigma), and 40 ng mL^−1^ dexamethasone. To induce hepatic steatosis in vitro, a 2:1 mixture of oleic acid and palmitic acid was added to AML12 cells for 24 h. Mouse recombinant irisin protein was expressed and purified using the pET bacterial expression system (Novagen) as previously described.^[^ ^77^ ^]^ For conditioned medium collection, differentiated C2C12 myotubes were transfected with siHSF1 or LV‐aHSF1 for HSF1 deficiency or activation. The conditioned medium was collected after transfection, cleared of cell debris by brief centrifugation, and applied to differentiated beige adipocytes and AML12 hepatocytes for 48 h. In parallel, cells were treated with recombinant irisin protein (1 µg mL^−1^), vehicle control, anti‐FNDC5 neutralizing antibody (1.25 mg mL^−1^, ab174833, Abcam), or normal rabbit IgG control (1.25 mg mL^−1^, 2729, CST) for 48 h to evaluate rescue effects. After treatment, differentiated C3H10T1/2 adipocytes were incubated with the thermogenic probe ERthermAC (250 nM, SCT057, EMD Millipore) for 30 min and analyzed by flow cytometry (BD LSRFortessa). Thermogenic activity was further assessed by quantitative PCR analysis of thermogenic marker genes. AML12 hepatocytes were stained with BODIPY 493/503 (D3922, Thermo Fisher Scientific) to visualize lipid accumulation and analyzed for the expression of de novo lipogenesis‐related genes.

### Oxygen Consumption Rate (OCR) Measurements

Differentiated C2C12 myotubes with indicated treatment were subjected to mitochondrial respiration measurements by using Seahorse XFe24 Extracellular Flux Analyzer (Agilent). One hour before the assay, the culture medium was replaced with Seahorse XF DMEM assay medium (103575‐100, Agilent) supplemented with 10 mM glucose (103577‐100, Agilent), 1 mM sodium pyruvate (103578‐100, Agilent), and 2 mM glutamine (103579‐100, Agilent), and the pH was adjusted to 7.4. The mitochondrial stress test was performed according to the manufacturer's instructions using the Seahorse XF Cell Mito Stress Test Kit (103015‐100, Agilent). Sequential injections of mitochondrial inhibitors were applied to achieve final concentrations of 1 µM oligomycin, 2 µM FCCP, and 0.5 µM rotenone/antimycin A. Oxygen consumption rate (OCR) was recorded at each measurement point using Seahorse Wave software (Agilent). After the assay, cells in each well were lysed in RIPA buffer, and total protein concentration was determined using the BCA protein assay kit (P0012, Beyotime). OCR values were normalized to total protein content.

### In Vivo and Ex Vivo Muscle Function Tests

The treadmill exhaustion test was conducted according to prior studies.^[^ ^78^ ^]^ Briefly, mice were subjected to adaptation to the treadmill (SA101, SANS Bio Instrument) for a week before the treadmill endurance test. After acclimation, the treadmill machine was started at a speed of 10 m min^−1^ for an initial hour with a 0‐degree inclination angle and increased by 2 m min^−1^ increments every 15 min until the mice remained stationary for the moderate electric stimulation (less than 0.1 mA) for more than 5s without resuming running. For the grip strength assessment, the whole limb muscular strength of mice was measured with a digital grip strength instrument (BIO‐GS3, BIOSEB Research Instruments) as previously reported.^[^ ^21^ ^]^ The tetanic contraction force of isolated EDL muscles was measured *ex vivo* in an oxygenated Krebs buffer according to the method previously described.^[^ ^79^ ^]^ Muscle contractions were triggered and recorded using a Dynamic Muscle Control system (ASI600A, Aurora Scientific) with the following stimulation parameters: a 0.5 s delay, 120 Hz frequency, and 0.3 s pulse duration.

### In Vivo Gene Delivery and Pharmacological Activation

Adeno‐associated virus 2/9 (AAV2/9) vector‐mediated overexpression of mouse HSF1 active form was packaged by Hanbio Technology Company (Shanghai, China) under the control of the CMV promoter, incorporating a Flag epitope tag.^[^ ^10^ ^]^ AAV‐GFP was used as a control. For local overexpression of HSF1 in skeletal muscles, AAV‐GFP or AAV‐active HSF1 (aHSF1) was intramuscularly injected into bilateral quadriceps (QU) and gastrocnemius (GAS) muscles of 22‐month‐old mice, with a viral titer of 5 × 10^10^ vector genomes (vg) in a final volume of 50 µl per muscle (2 × 10^11^ vg mice^−1^) for further analysis.^[^ ^21^, ^80^ ^]^

For in vivo genetic rescue, 18‐month‐old WT and HSF1‐HKO mice received intramuscular injection of AAV‐dMCK‐SIRT3 or AAV‐dMCK‐Con (5 × 10^10^ vg muscle^−1^, 2 × 10^11^ vg mice^−1^) into the bilateral QU and GAS muscles, forming three experimental groups: 1) WT + AAV‐Con; 2) HSF1‐HKO + AAV‐Con; (3) HSF1‐HKO + AAV‐SIRT3. After 6 weeks, mice were subjected to treadmill endurance and grip strength tests for functional evaluation. In a parallel cohort, 18‐month‐old WT and HSF1‐HKO mice were administered HKL (5 mg kg^−1^) or vehicle via intraperitoneal injection once daily for two weeks, followed by treadmill endurance and grip strength assessments.

For pharmacological activation of HSF1, mice were treated with celastrol (3 mg kg^−1^) according to previously described protocols.^[^ ^12^ ^]^ 18‐month‐old mice were fed standard chow with or without celastrol supplementation for 8 weeks. Celastrol was thoroughly mixed with powdered chow before pelleting to ensure uniform dosing. At the end of the treatment, food intake, grip strength, body composition, muscle weights, and molecular parameters of skeletal muscle were measured for further analysis.

### Echocardiography

Echocardiography was conducted using a Vevo 2100 system (VisualSonics) as previously described.^[^ ^81^ ^]^ Briefly, mice were anesthetized with 1.5–2% isoflurane and positioned supinely to allow for spontaneous breathing. M‐mode echocardiographic images were obtained at the level of the papillary muscles, and standard parameters, including fractional shortening (FS) and ejection fraction (EF), were measured using the system's software tools. These measurements were derived from the M‐mode traces to assess cardiac function. The echocardiographer was blind to the animal groups.

### Metabolic Analysis in Mice

To study glucose metabolism, glucose and insulin tolerance tests were performed as described previously.^[^ ^82^ ^]^ For the glucose tolerance test, mice were fasted for 6 h and injected intraperitoneally with a glucose solution dissolved in PBS (1.5 g kg^−1^ body weight, G8270, Sigma). For the insulin tolerance test, mice received an intraperitoneal injection of insulin at a dose of 0.75 U/kg body weight (I9278, Sigma). Glucose level was determined in tail blood at 0, 15, 30, 60, 90, and 120 min after injection, using an automatic glucometer (OneTouch Verio Flex, Johnson's). A concentration‐time curve was plotted, and the area under the curve (AUC) was calculated by GraphPad Prism 8 software.

For the quantification of hepatic triglyceride content, liver tissues were homogenized in 5% NP40 in PBS. Following centrifugation of the tissue lysates, the supernatant was collected. Triglyceride (TG) levels were then assessed using the TG Assay Kit (A110‐1‐1, NJJCBIO) and normalized to liver protein content using the BCA protein assay kit (Beyotime, P0012).

Serum parameters were measured via colorimetric assays with commercially available kits, including serum TG, total cholesterol (TC, A111‐1‐1, NJJCBIO), high‐density lipoprotein cholesterol (HDL, A112‐1‐1, NJJCBIO), and low‐density lipoprotein cholesterol (LDL, A113‐1‐1, NJJCBIO).

### Immunofluorescence Staining

C2C12 myotubes were washed with PBS and fixed with 4% Paraformaldehyde (PFA) in PBS for 15 min at room temperature (RT). After being washed three times, myotubes were permeabilization with PBS containing 0.2% Triton X‐100 for 15 min and then washed three times with PBS, followed by blocking for 2 h in PBS with 5% goat serum. Myotubes were incubated with mouse anti‐myosin (MF‐20, DSHB) or rabbit anti‐TOM20 (A19403, ABclonal) diluted 1:200 in PBS with 5% goat serum at 4 °C overnight. The cells were washed three times with PBS containing 0.1% Tween 20 (PBST) and then incubated with Alexa Fluor 488‐conjugated AffiniPure goat anti‐mouse IgG (H+L) (115‐545‐003, Jackson ImmunoResearch) or Alexa Fluor 594‐conjugated AffiniPure goat anti‐rabbit IgG (H+L) (111‐585‐003, Jackson ImmunoResearch) for 2 h at RT. In addition, the nuclei were stained with Hoechst 33342 (C1027, Beyotime) for 10 min. Myotube cultures were visualized under a fluorescence microscope (ECLIPSE Ts2, Nikon) at 100X magnification. Myotube diameters were quantified by measuring a total of 200 myotube diameters from 10 random fields of view using Image‐Pro Plus 6.0 software. Results were expressed as the fold change in diameter relative to the control group.

### Plasmid Constructions, Transfections, and Luciferase Assays

The active form of HSF1 plasmid was constructed as previously reported.^[^ ^10^ ^]^ The *Sirt3* and *Hsf1* luciferase reporter and the HSE or E‐box deletion reporter were cloned into a pGL4.17‐basic vector. All constructs were confirmed by DNA sequencing. Primer pairs for plasmid construction are listed in Table S2 (Supporting Information). HEK293T cells were transfected with indicated plasmids using an EZ‐trans transfection reagent (Life Ilab Biotechnology, C4058L1090). After transfection, luciferase activity was determined by a dual luciferase system (Promega, TM040).

### RNA Extraction, qRT‐PCR, and RNA‐seq

Total RNA was isolated from tissues or cultured cells using RNAiso Plus (9108, Takara) and reverse transcribed to cDNA with PrimeScript RT Master Mix (RR036A, TaKaRa) according to the manufacturer's instructions. The mRNA expression levels were quantified by quantitative real‐time PCR by using SYBR Green Master mix (11143ES50, Yeasen) on a Roche Light Cycler 480 system (Roche). The results were calculated by the 2^−ΔΔCT^ method, followed by normalization to *36B4* or *Gapdh* expression as appropriate for the experimental context. Details of the primer sequences are listed in Table S3 (Supporting Information).

For RNA‐seq, GAS muscles from WT and MKO mice were used for RNA extraction. RNA quality was assessed using the RNA Nano 6000 Assay Kit of the Bioanalyzer 2100 system (Agilent Technologies) and agarose gel electrophoresis, followed by cDNA library construction. mRNA was purified from total RNA using poly‐T oligo‐attached magnetic beads and fragmented. The cDNA library preparation included end repair, A‐tailing, adapter ligation, size selection, amplification, and purification, resulting in a ready‐to‐sequence library. Subsequently, the library was quantified by Qubit for clustering and sequencing on an Illumina Novaseq platform. Clean RNA‐seq data were obtained by filtering out reads containing adapters, poly‐N regions, and those with low‐quality scores, followed by mapping to the reference genome GRCm39 using Hisat2 (v2.0.5). The gene expression levels were quantified with Feature Counts (v1.5.0‐p3), followed by differential expression analysis with the DESeq2 R package (v1.20.0), where genes with an adjusted *p*‐value < 0.05 and |FC| > 2 were considered differentially expressed (DEGs). Gene ontology (GO) and KEGG enrichment analyses were performed in the DAVID bioinformatics resource (https://david.ncifcrf.gov).

To identify Fndc5/Irisin, a list of down‐regulated genes from the RNA‐seq analysis was intersected with a list of secreted proteins obtained from the Secreted Protein Database (SEPDB).^[^ ^41^ ^]^ The genes were then ranked based on their adjusted p‐values derived from the RNA‐seq dataset, with the top‐ranked genes being selected for further assessment.

### ChIP and ChIP‐Seq

Differentiated C2C12 myotubes were transfected with Lentivirus‐mediated HSF1 overexpression for two days, and then harvested for ChIP assays. ChIP experiments were performed using a SimpleChIP Enzymatic Chromatin IP Kit (9003, CST) according to the manufacturer's instructions. In brief, cells were cross‐linked by 1% formaldehyde for 10 min and quenched by glycine. The extracted chromatin was performed with sonication, immunoprecipitation by anti‐HSF1 (4356S, CST) and normal IgG (2729, CST) using Magnetic beads, reverse cross‐linking and DNA purification. Immunoprecipitated DNA was quantified by qPCR. ChIP primers are listed in Table S4 (Supporting Information).

Library preparation and sequencing were conducted by Novogene Biotechnology (Shanghai, China) using the Illumina platform. Raw reads were processed with fastp (v0.19.11) and aligned to the reference genome using BWA mem (v0.7.12). MACS2 (v2.1.0) was used for peak calling, and results were visualized with the WashU Epigenome Browser. A combinatorial analysis was conducted using a dataset of ≈5000 HSF1‐bound genes in C2C12 myotubes and down‐regulated mRNA expression observed in the GAS muscle of HSF1 MKO models, resulting in a distinct set of genes directly regulated by HSF1. Subsequently, Reactome pathway enrichment analysis was performed with these HSF1 direct targets (68 genes) as input in the DAVID bioinformatics resource.

### Co‐IP Assay

For the co‐immunoprecipitation (Co‐IP) of PGC1α1, PGC1α4 and SIRT3, HEK293T cells were allocated into four distinct transfection groups: (1) GFP and SIRT3; (2) GFP‐fused PGC1α1 and SIRT3; (3) GFP‐fused PGC1α4 and SIRT3. The construction of these plasmids has been previously reported in the studies.^[^ ^21^ ^]^ After 48 h of transfection, HEK293T cells and GAS muscles from indicated groups were lysed in IP lysis buffer (P0013, Beyotime) and centrifuged at 12,000 rpm for 20 min at 4 °C. The supernatant was then incubated with protein A/G PLUS‐agarose (sc‐2003, Santa Cruz), anti‐GFP (50430‐2‐AP, Proteintech), anti‐PGC1α (sc‐518025, Santa Cruz) and anti‐SIRT3 (A20805, ABclonal) overnight at 4 °C. The protein samples were washed, eluted, and boiled with SDS loading dye buffer at 100 °C for 10 min for subsequent western blot analysis.

For endogenous Co‐IP analysis, 20–30 µL of resuspended volume of protein A/G PLUS‐agarose was pre‐incubated with 2 µg per sample of anti‐PGC1α antibody (sc‐518025, Santa Cruz) against the N‐terminus of PGC1α that recognizes PGC1α1 and PGC1α4 as described previously for 3 h at 4 °C.^[^ ^42^ ^]^ C2C12 myotubes and GAS muscles from indicated groups were lysed in IP lysis buffer and centrifuged to obtain the supernatant. Then, the 500 µg of protein extract was added to the pre‐incubation mixture and incubated on a rotator overnight at 4 °C. Affinity beads were washed 3 times, followed by western blot analysis.

### Protein Extraction and Western Blot

Samples were homogenized and lysed in RIPA buffer (P0013B, Beyotime) supplemented with 1 mM PMSF, 10 mM DTT, and 10 µM protein kinase inhibitor on ice for 15 min. The nuclear and cytoplasmic fractions were prepared with Nuclear and Cytoplasmic Protein Extraction Kit (P0028, Beyotime) as described previously.^[^ ^21^ ^]^ Protein concentrations were quantified via a BCA Protein quantification kit (P0010, Beyotime), and equal protein were loaded onto SDS‐PAGE gel for separation and transferred onto nitrocellulose (NC) membranes. After nonspecific sites were blocked with 5% skimmed milk or bovine serum albumin (BSA) for 1–2 h at room temperature. Primary antibodies against HSF1 (GB11931‐100, Servicebio), p‐HSF1‐Ser326 (bsm‐52166R, Bioss), MAFbx (sc‐166806, Santa Cruz), MuRF‐1 (sc‐398608, Santa Cruz), MSTN (sc‐393335, Santa Cruz), IGF1 (sc‐74116, Santa Cruz), p‐mTOR‐Ser2448 (2971S, CST), t‐mTOR (AM832, Beyotime), p‐S6K1‐Thr389 (9205S, CST), t‐S6K1 (9202S, CST), PGC1α (sc‐518025, Santa Cruz), p‐FAK‐Tyr397 (AF5821, Beyotime), t‐FAK (AF1108, Beyotime), p‐AKT‐Thr308 (AF5734, Beyotime), t‐AKT (AA326, Beyotime), p‐CREB‐Ser133 (AG1680, Beyotime), t‐CREB (AF1018, Beyotime), OXPHOS Rodent WB Antibody Cocktail (ab110413, Abcam), CS (A5713, ABclonal), SIRT3 (A20805, ABclonal), β‐actin (sc‐8432, Santa Cruz) and GAPDH (GB15002‐100, Servicebio) were applied, followed by incubation with appropriate secondary antibodies. Band detection and quantification were carried out using the Odyssey imaging system (LI‐COR Biotechnology) and ImageJ software, respectively.

### Histological Analysis

Freshly isolated muscle tissues were embedded in OCT and snap‐frozen cold isopentane. Adipose and liver tissues were fixed with a 10% neutral formaldehyde solution and processed into paraffin blocks following standard protocols. 10 µm transverse cryosections from muscles were cut from the mid‐belly area and conducted with H&E and succinate dehydrogenase (SDH) staining. Meanwhile, 4 µm paraffin sections from adipose and liver tissues were cut and stained with H&E. Tissue sections were photographed using a Nikon camera. For the morphological assessments via H&E staining, including myofiber sizes and the number of myofibers with tubular aggregates or central nuclei, more than 500 myofibers from 5 random fields of view in GAS and QU were analyzed using Image‐Pro Plus 6.0 software. To assess mitochondrial content and function in the skeletal muscle, SDH staining was performed in the gastrocnemius/plantaris (GAS/PL) muscle complex. The intensity of SDH‐positive fibers was evaluated in 5 randomly selected fields of view from the PL, RG, and WG muscle regions, respectively. For immunofluorescence staining, the following antibodies were used: MYH7 (slow, GB121857, Servicebio), MYH4 (fast, 20140‐1‐AP, Proteintech), Laminin (sc‐59854, Santa Cruz), PAX7 (DSHB, PAX7), CD140a (PDGFRA, 14‐1401‐82, eBioscience) and appropriate fluorophore‐conjugated secondary antibodies. Fluorescent images were acquired using a Leica TCS SP8 confocal microscope. Slow/fast fiber cross‐sectional area and number were quantified using Cellpose and ImageJ (Fiji) software.^[^ ^83^ ^]^

### Transmission Electron Microscopy (TEM) Analysis

QU muscles (1 mm^3^) were fixed with 2.5% glutaraldehyde at 4 °C, followed by post‐fixation in 1% OsO4. After washing with 0.1 M phosphate buffer (PB) and dehydration in a graded series of ethanol, the samples were infiltrated with 100% acetone and embedded in Epon 812. Polymerization was performed at 37 °C for 18 h, followed by 48 °C for 24 h, and 60 °C for 48 h. Ultrathin sections (70 nm) were cut and stained with uranyl acetate and lead citrate for analysis using a JEM2100 transmission electron microscope (JEOL, Japan) at the Electron Microscopy Center of East China Normal University. Mitochondrial analysis was conducted according to the method previously described.^[^ ^84^ ^]^ Briefly, intermyofibrillar (IMF) and subsarcolemmal (SS) mitochondria populations from each section were photographed in more than 5 fields of view. The number and area of IMF and SS mitochondria were counted and measured in each group using Image‐Pro Plus 6.0 software, respectively.

### Measurement of ATP Contents

ATP content in skeletal muscle and C2C12 myotubes was measured using a firefly luciferase‐based ATP assay kit (S0027, Beyotime) according to the manufacturer's protocol. Briefly, samples were lysed and centrifuged at 10000 × g for 15 min at 4 °C. The supernatant was then incubated with the ATP reaction mix for 5 min, and chemiluminescence was detected using a fluorescence microplate reader. ATP contents were determined from a standard curve and normalized to protein concentrations.

### Statistical Analysis

Statistical analysis was performed with GraphPad Prism 8 software. The statistical details of the experiment are indicated in the figure legend. The normality of data was examined by the Shapiro‐Wilk normality test. Two‐tailed unpaired Student's *t*‐test was used for statistical comparisons between the two groups. One‐way ANOVA with Tukey's multiple comparisons or two‐way ANOVA with Bonferroni's post hoc test was used for comparisons between multiple groups. ANCOVA analysis by SPSS software was used for comparisons of oxygen consumption and carbon dioxide production with body weight as a covariate. The correlation between *HSF1* mRNA levels and human age was analyzed by Pearson correlation analysis. All data were presented as mean ± SEM. *p* < 0.05 was considered as statistically significant, *p < 0.05, **p < 0.01, ns, not significant. The schematic illustration was created with BioRender.com.

## Conflict of Interest

The authors declare no conflict of interest.

## Author Contributions

J.Z., M.H., X.W., and M.G. contributed equally to this work. L.X., X.M., and C.H. devised and supervised the project. J.Z., M.H., X.W., M.G., Y.M., S.W., F.C., and Y.Z. performed animal experiments. J.Z., M.H., X.W., Y.M., and Y.C. performed biochemical and cellular experiments. W.W., Y.L., Y.Z., J.X., and Z.G. provided additional support for the animal and cellular experiments. Y.W. and J.Q. prepared the samples for TEM and analyzed electron micrographs. C.H. collected human muscle biopsies, and J.Z. performed data analysis. L.X., X.M., and J.Z. wrote and edited the manuscript. All authors have read and approved the manuscript.

## Supporting information



Supporting Information

## Data Availability

The data that support the findings of this study are available from the corresponding author upon reasonable request.
