# Supplementary material for: Skeletal Muscle HSF1 Alleviates Age‐Associated Sarcopenia and Mitochondrial Function Decline via SIRT3‐PGC1α Axis
Source: Adv Sci (Weinh). 2025 Dec 16;13(11):e10368. doi: 10.1002/advs.202510368 (PMC12931205; doi:10.1002/advs.202510368)
Supplement: Supplementary file 1 — Supporting Information [file ADVS-13-e10368-s001.pdf]

Supporting Information

Skeletal Muscle HSF1 Alleviates Age-associated Sarcopenia and Mitochondrial Function Decline via SIRT3-PGC1α Axis

Jun Zhang, Min Hu, Xia Wu, Mingwei Guo, Ying Ma, Jin Qiu, Siqi Wang, Yuxiang Cao, Yinzhao Zhong, Fangfang Chen, Yiwen Wang, Wei Wei, Yan Lu, Yong Zhang, Junjie Xiao, Zhenji Gan, Cheng Hu, Xinran Ma, Lingyan Xu

This PDF file includes:

Figure S1 to S14

Table S1 to S4

Figure S1

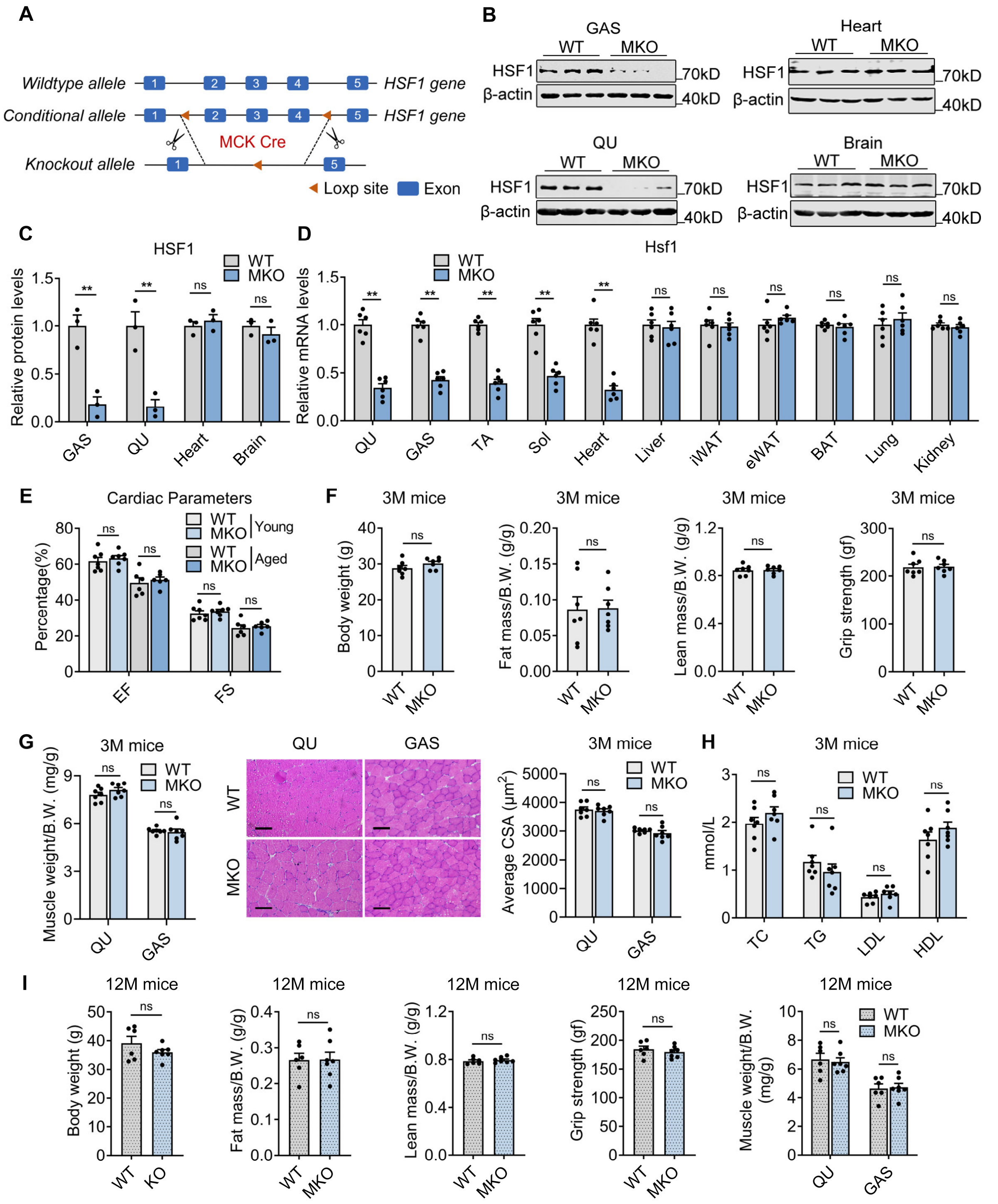

Figure S1. Muscle-specific HSF1 knockout may not impact muscle development or cardiac function in young and aged mice

(A-D) Strategy for generating *Hsf1*$^{f/f}$;*MCK-Cre* (HSF1-MKO) mice, with HSF1 expression levels in aged (22-month-old) skeletal muscle and other tissues. (E) Heart function was evaluated by ejection fraction (EF) and fractional shortening (FS) using echocardiography in young and aged mice of the indicated genotypes. (F) Body weight, body composition, and grip

strength of young (3-month-old) male mice. (G) Muscle weights, representative H&E staining, and quantifications of myofiber sizes. (H) Serum parameters in young mice of the indicated genotypes. (I) Body weight, body composition, grip strength, and muscle weights of middle-aged (12-month-old) male mice. Scale bar, 100 μm. 3-month group, n = 7; 12-month group, n = 6-7; 22-month group, n = 6.

Statistical significance was assessed by unpaired Student's t test (C-I). Data are presented as mean ± SEM and $^{**}P<0.01$; ns, not significant compared to control group.

Figure S2

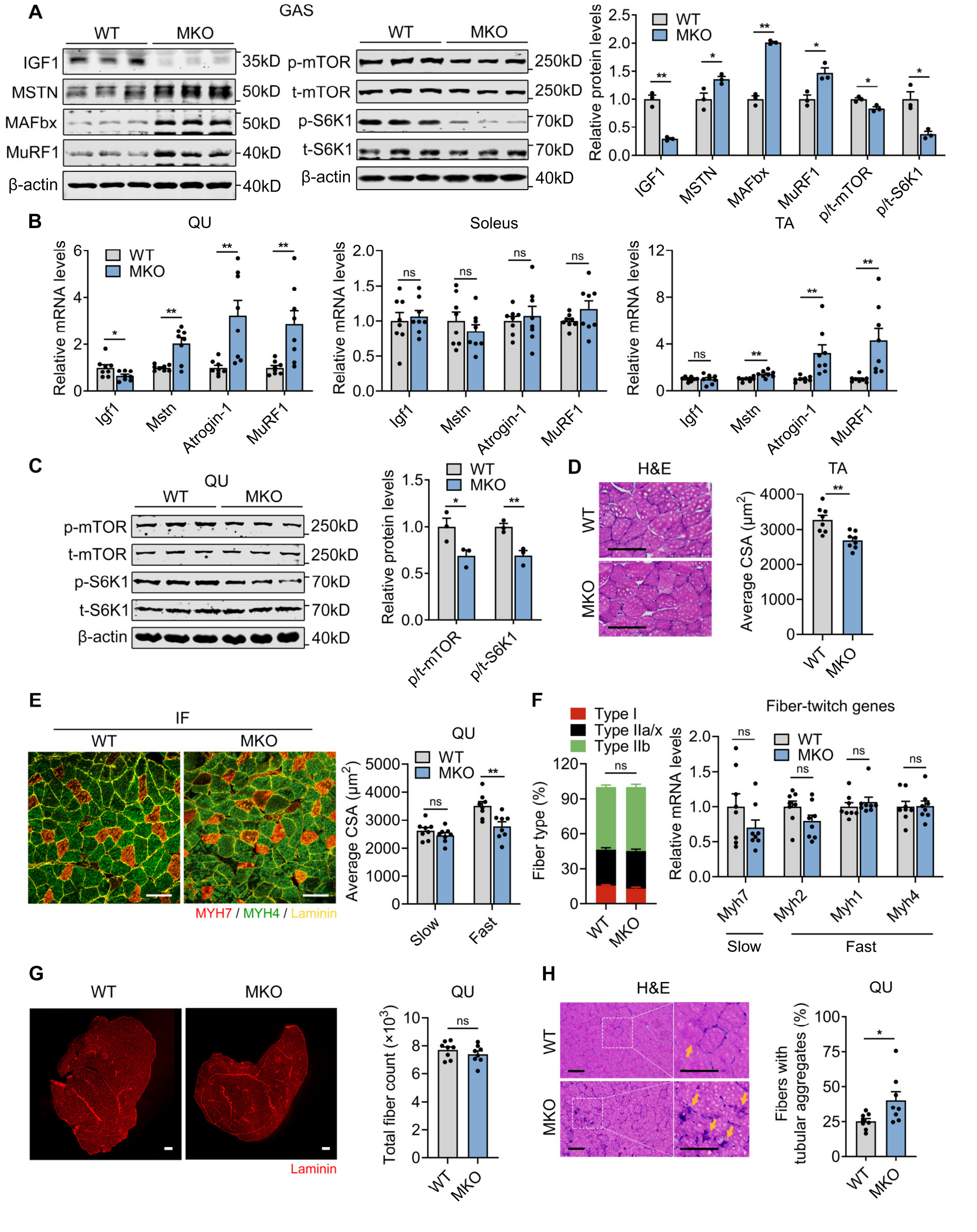

Figure S2. Muscle HSF1 deficiency exacerbates age-related muscle atrophy and weakness in aged mice

(A) Representative western blot analysis and quantification of protein levels of muscle atrophic markers and mTOR-S6K1 in GAS muscles of indicated genotypes (n=3). (B) mRNA levels of atrophic genes in QU, TA and soleus muscles of the indicated genotypes. (C) Representative western blot analysis and quantification of mTOR-S6K1 in QU muscles of indicated genotypes

(n=3). (D) Representative H&E staining and quantifications of fiber sizes in TA muscles of the indicated genotypes. (E) Representative MyHC IF staining in QU muscles of indicated genotypes and quantification of fiber size of slow- and fast-twitch fibers. (F) Fiber-type composition in red GAS muscles and mRNA expression of the corresponding MyHC isoforms (I, IIa, IIx, IIb) of the indicated genotypes. (G) Representative Laminin IF staining in QU muscles of indicated genotypes and quantification of total fiber number. (H) Representative H&E staining and quantifications of fibers with tubular aggregates in QU muscles of the indicated genotypes. Yellow arrows indicate myofibers with tubular aggregates. n=8 mice per group unless otherwise noted. Scale bar represents 100 μm.

Statistical significance was assessed by unpaired Student's t test (A-H) and two-way ANOVA with Bonferroni's post hoc test (F). Data are presented as mean ± SEM and *$P<0.05$; **$P<0.01$; ns, not significant compared to control group.

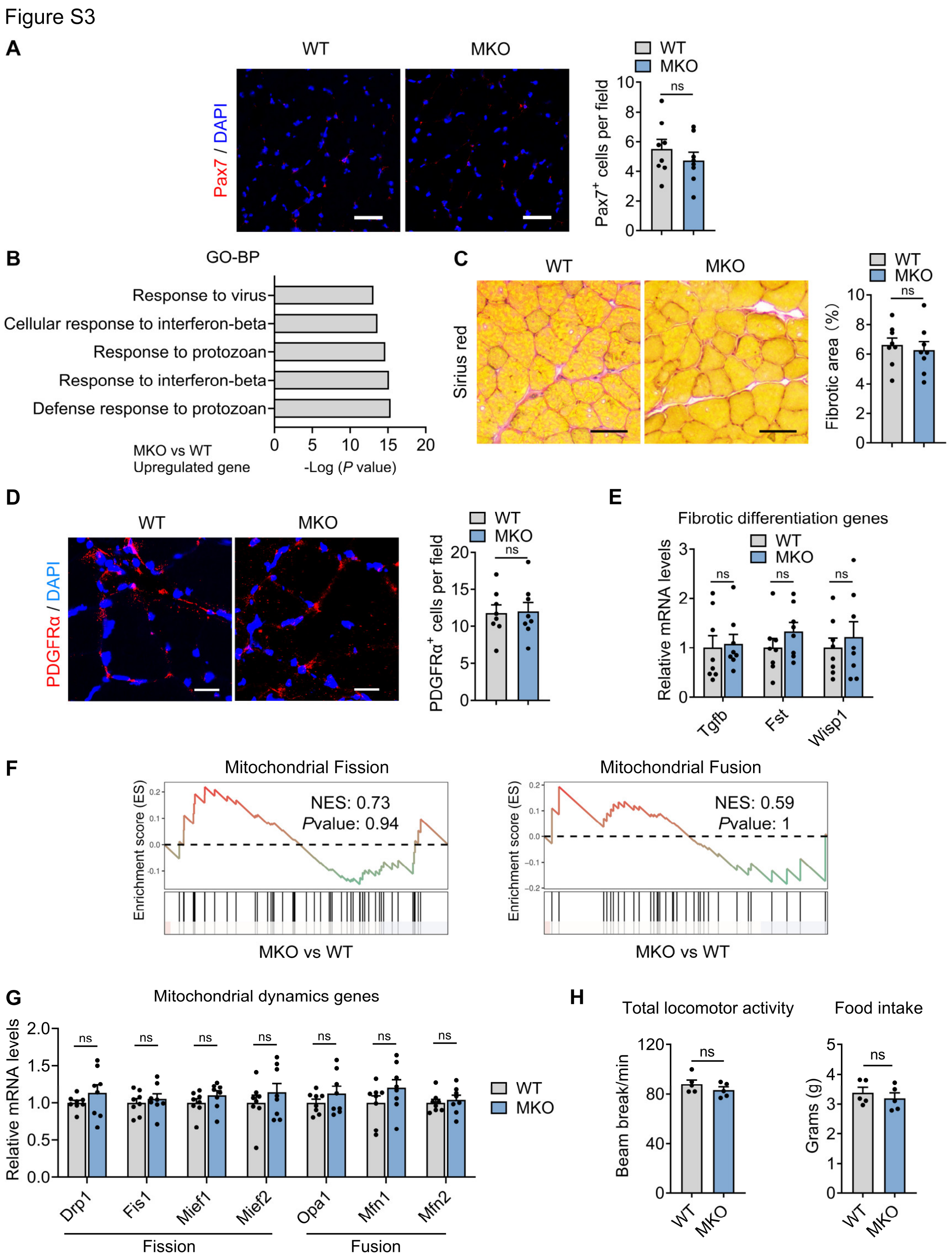

Figure S3. Assessment of muscle fibrosis, satellite cell pool, and metabolic profile in HSF1-MKO mice

(A) Representative Pax7 IF staining and quantification of Pax7$^+$ satellite cells in aged muscles of indicated genotypes. Scale bar, 50 μm. (B) GO enrichment analysis of upregulated DEGs in HSF1-MKO muscles. (C) Representative sirius red staining and quantification of fibrotic area in QU muscles of indicated genotypes. Scale bar, 50 μm. (D) Representative PDGFRα IF

staining and quantification of PDGFRα$^+$ cell numbers in QU muscles of indicated genotypes. Scale bar, 20 μm. (E) mRNA levels of FAP differentiation in QU muscles of indicated genotypes. (F) GSEA analysis of mitochondrial fission and fusion pathways. (G) mRNA levels of genes involved in mitochondrial dynamics of indicated genotypes. (H) total locomotor activity and food intake of indicated genotypes (n=5). n=8 mice per group unless otherwise noted.

Statistical significance was assessed by unpaired Student's t test (B-G). Data are presented as mean ± SEM and ns, not significant compared to control group.

Figure S4

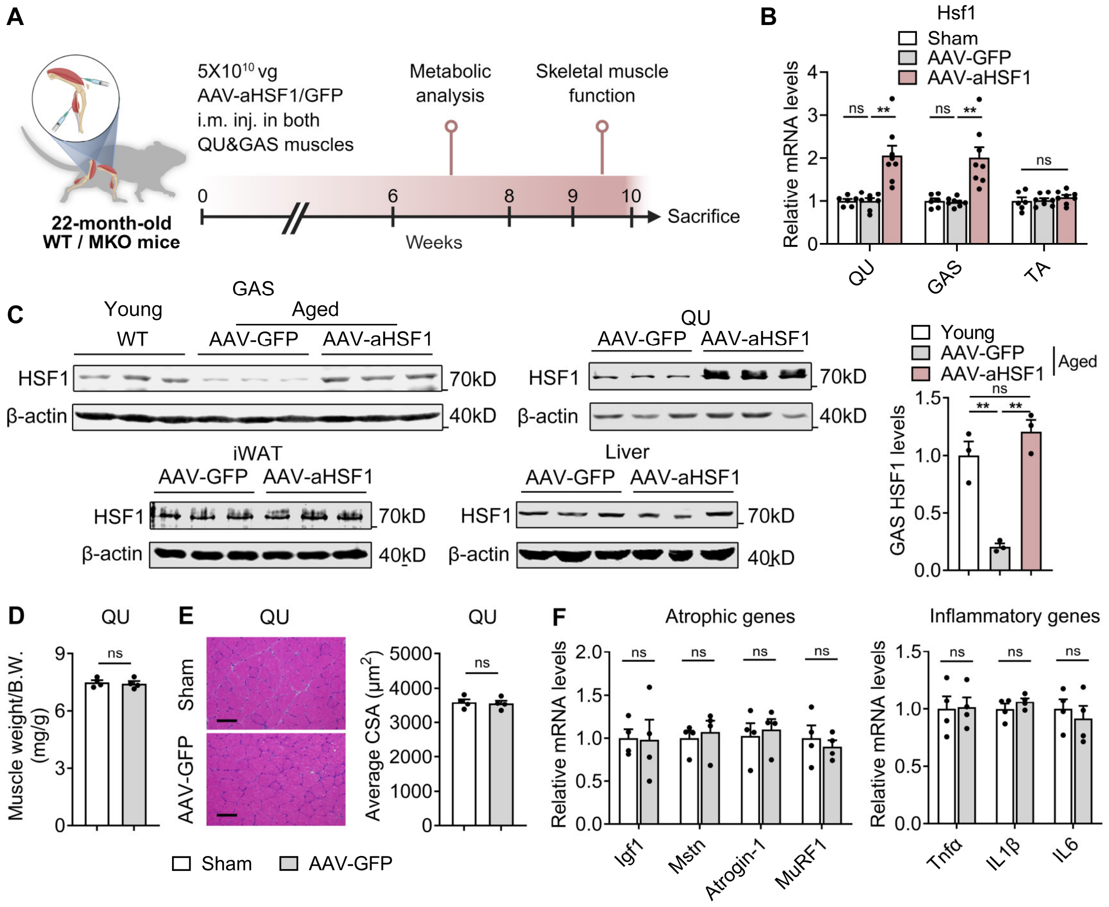

Figure S4. Active HSF1 overexpression ameliorates sarcopenia in aged muscle with non-injected and contralateral controls

(A) Schematic illustration of AAV-mediated active form of HSF1 overexpression in QU and GAS muscles of aged (22-month-old) mice. (B) mRNA levels of *Hsf1* in muscles of the indicated groups (n=6-8). (C) Representative western blot analysis of HSF1 protein levels in muscles and other tissues of the indicated groups (n=3). (D) Muscle weights of the indicated groups. (E) Representative H&E staining and quantifications of fiber sizes in QU muscles of the indicated groups. Scale bar represents 100 μm. (F) mRNA levels of atrophic and inflammatory genes in QU muscles of the indicated groups (n=4).

Statistical significance was assessed by one-way ANOVA with Tukey's multiple comparisons (B and C) and unpaired Student's t test (D-F). Data are presented as mean ± SEM and $^{**}P<0.01$; ns, not significant compared to control group.

Figure S5

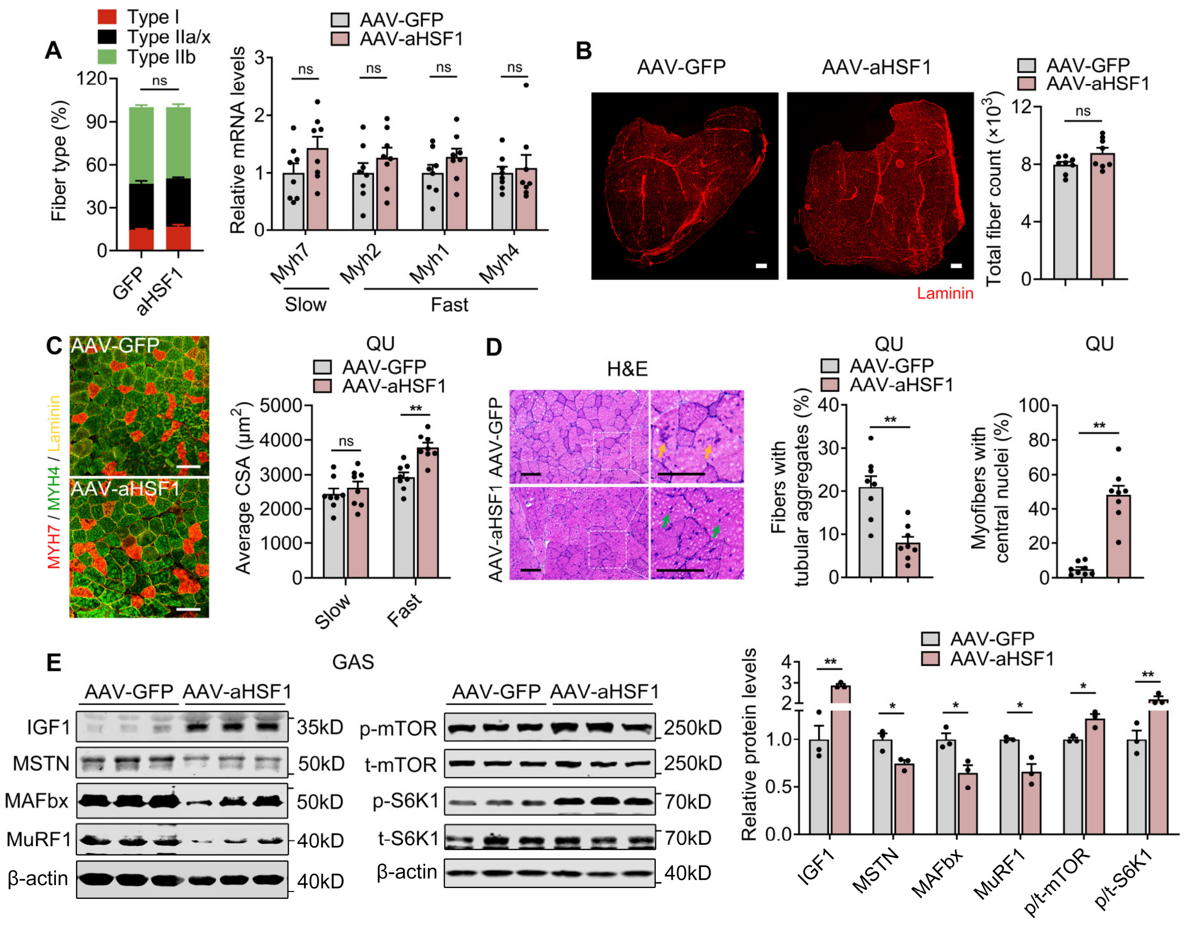

Figure S5. Active form of HSF1 overexpression improves sarcopenia and muscle functions (A) Fiber-type composition in red GAS muscles and mRNA expression of the corresponding MyHC isoforms (I, IIa, IIx, IIb) of the indicated groups. (B) Representative Laminin IF staining in QU muscles of indicated groups and quantification of total fiber number. (C) Representative MyHC IF staining in QU muscles of indicated groups and quantification of fiber size of slow- and fast-twitch fibers. (D) Representative H&E staining and quantifications of fibers with tubular aggregates and central nuclei in QU muscles of the indicated groups. (E) Representative western blot analysis and quantification of muscle atrophic markers and mTOR-S6K1 in GAS muscles of indicated groups (n=3). n=8 mice per group unless otherwise noted. Scale bar represents 100 μm.

Statistical significance was assessed by two-way ANOVA with Bonferroni's post hoc test (A) and unpaired Student's t test (A-E). Data are presented as mean ± SEM and **P*<0.05; ***P*<0.01; ns, not significant compared to control group.

Figure S6

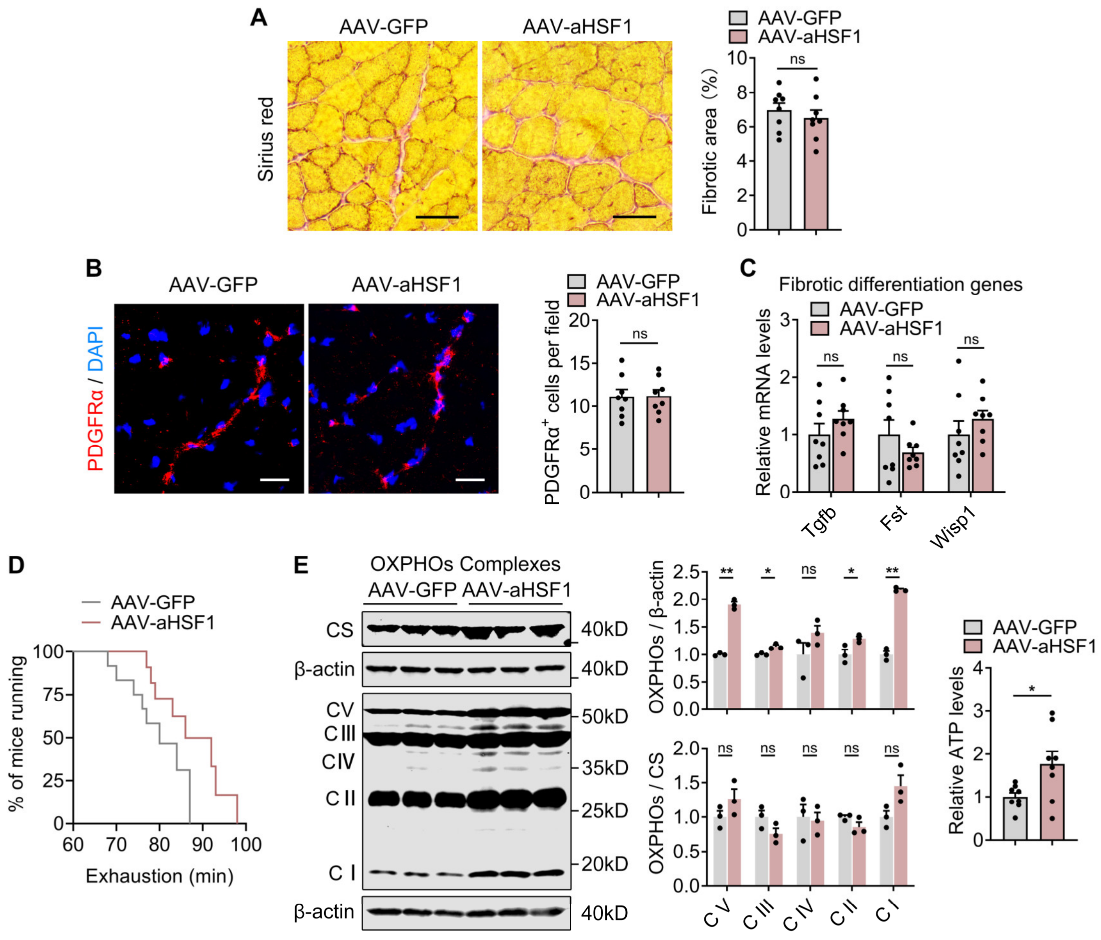

Figure S6. Effects of AAV-aHSF1 treatment on fibrosis and mitochondrial function

(A) Representative sirius red staining and quantification of fibrotic area in QU muscles of the indicated groups. Scale bar, 50 μm. (B) Representative PDGFRα IF staining, quantification of PDGFRα$^+$ cell numbers of the indicated groups. Scale bar, 20 μm. (C) mRNA levels of FAP differentiation in QU muscles of the indicated groups. (D) Running population plotted against time to exhaustion of the indicated groups. (E) Representative western blot analysis, quantification of OXPHOS complexes (n=3; normalized to citrate synthetase), and ATP levels in GAS muscles of the indicated groups. n=8 mice per group unless otherwise noted.

Statistical significance was assessed by unpaired Student's t test (A-C and E). Data are presented as mean ± SEM and *P<0.05; ns, not significant compared to control group.

Figure S7

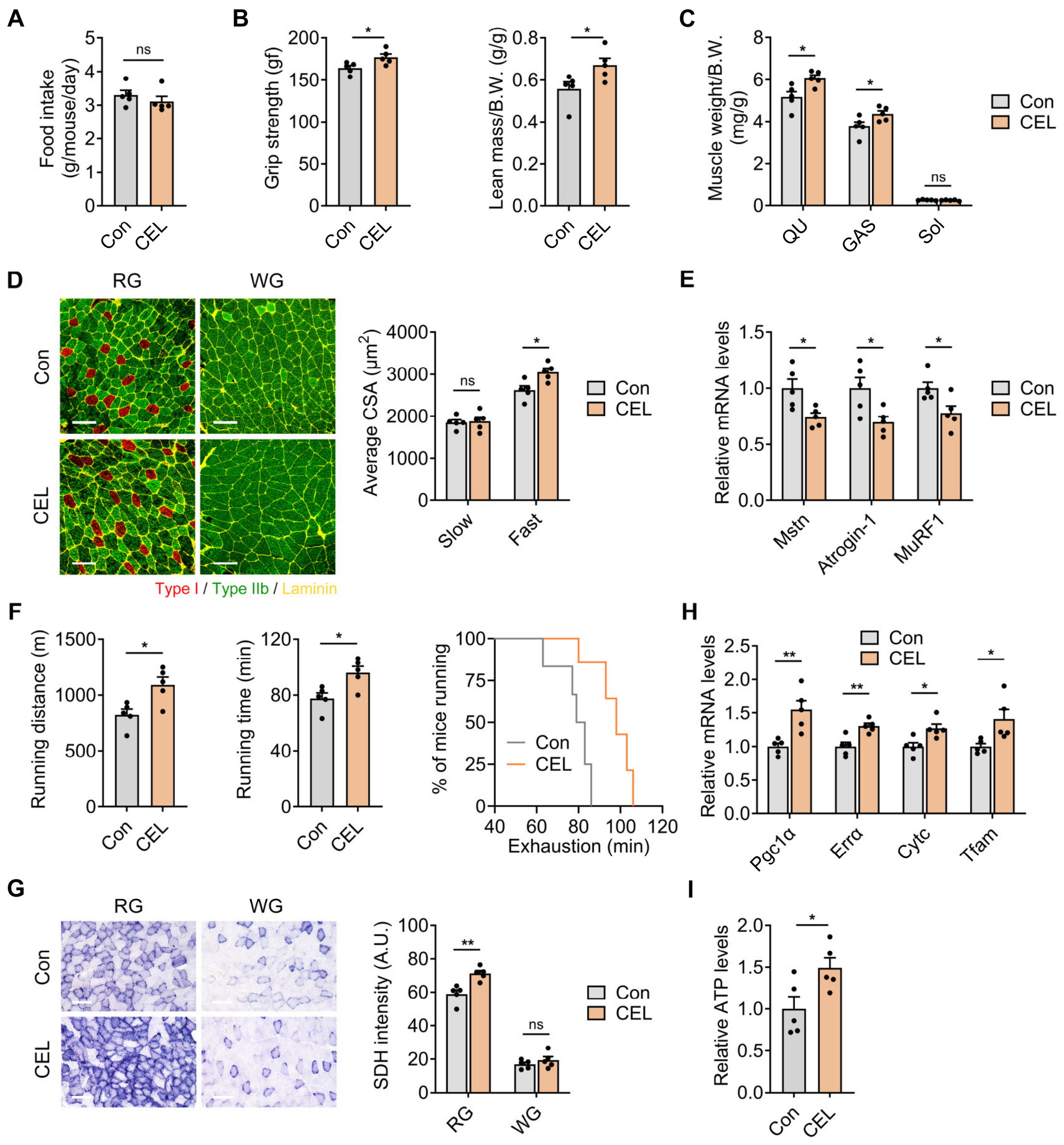

Figure S7. Celastrol-mediated HSF1 activation protects against age-associated sarcopenia (A) food intake, (B) grip strength, normalized lean mass, and (C) muscle weights of the indicated groups. (D) Representative MyHC IF staining in GAS muscles and quantification of fiber size of slow- and fast-twitch fibers of the indicated groups. (E) mRNA levels of atrophic genes in GAS muscles. (F) Treadmill endurance performance. (G) Representative SDH staining, quantification of SDH intensity of the indicated groups. (H) mRNA levels of mitochondrial genes in GAS muscles. (I) ATP levels in GAS muscles of the indicated groups. Scale bar represents 100 μm. n=5 mice per group unless otherwise noted.

Statistical significance was assessed by unpaired Student's t test (A-I). Data are presented as mean ± SEM and *P<0.05; **P<0.01; ns, not significant compared to control group.

Figure S8

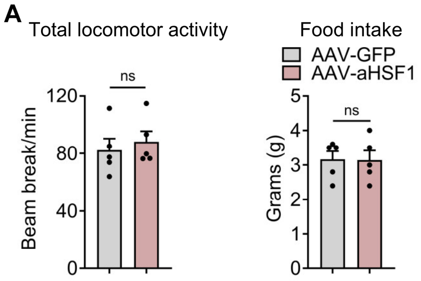

Figure S8. Metabolic parameters of mice with muscle HSF1 overexpression

(A) Total locomotor activity and food intake of indicated groups. n=5 mice per group. Statistical significance was assessed by unpaired Student's t test. Data are presented as mean ± SEM and ns, not significant compared to control group.

Figure S9

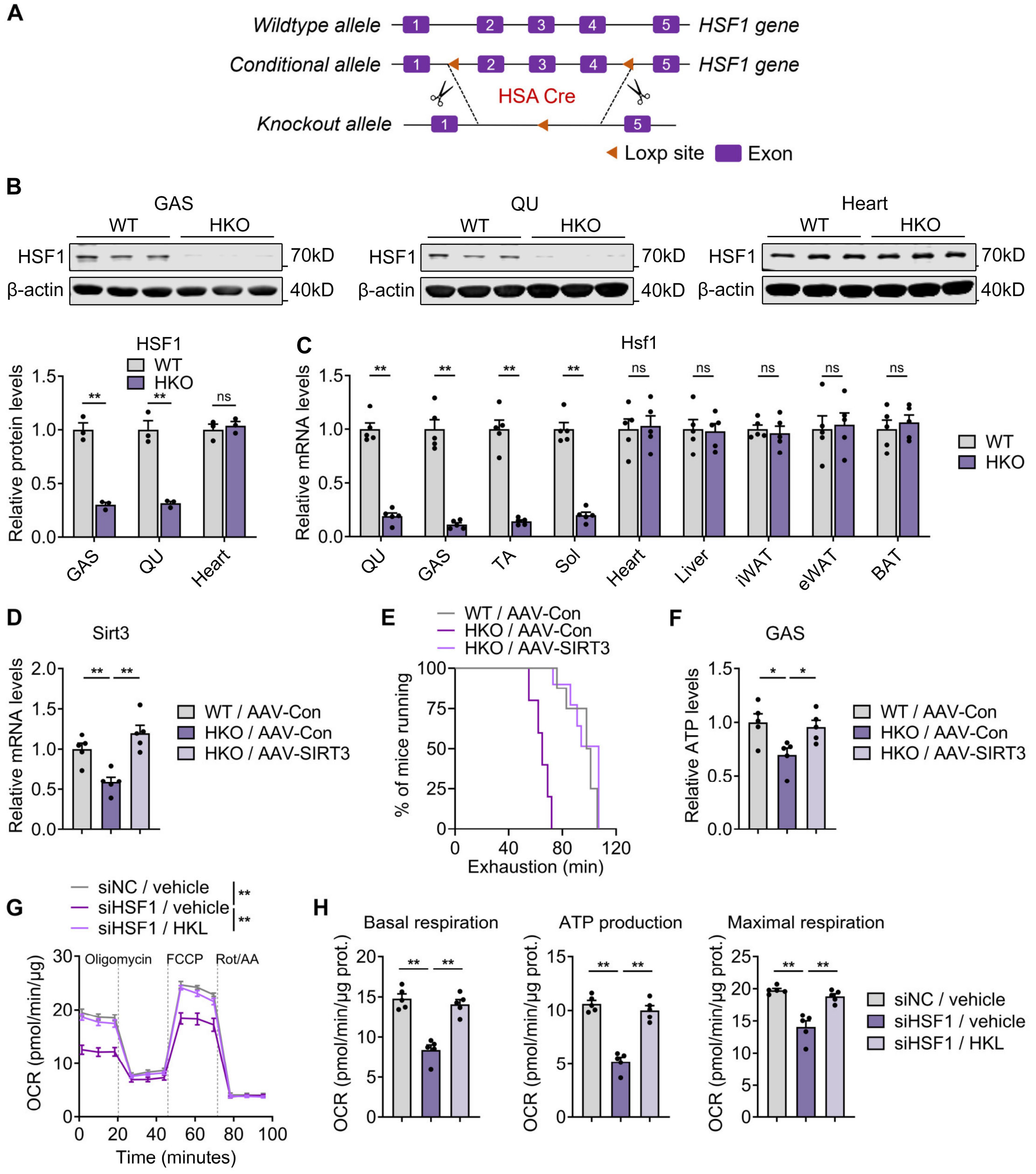

Figure S9. AAV-mediated SIRT3 overexpression restores the HSF1-deficient phenotypes *in vivo*

(A-C) Strategy for generating *Hsf1$^{f/f}$*; *HSA-Cre* (HSF1-HKO) mice, with HSF1 expression levels in aged (18-month-old) skeletal muscle and other tissues. (D) *Sirt3* mRNA levels in GAS muscles of the indicated groups. (E) Running population plotted against time to exhaustion of the indicated groups. (F) ATP levels in GAS muscles of the indicated groups. (G) Oxygen consumption rate (OCR) traces in C2C12 myotubes with SIRT3 rescue. (H) Quantification of basal respiration, ATP production, and maximal respiration in C2C12 myotubes in response to HKL. n=5 mice per group unless otherwise noted.

Statistical significance was assessed by unpaired Student's t test (B and C), one-way ANOVA with Tukey's multiple comparisons (D, F and H) and two-way ANOVA with Bonferroni's post hoc test (G). Data are presented as mean ± SEM and *P<0.05; **P<0.01; ns, not significant compared to control group.

Figure S10

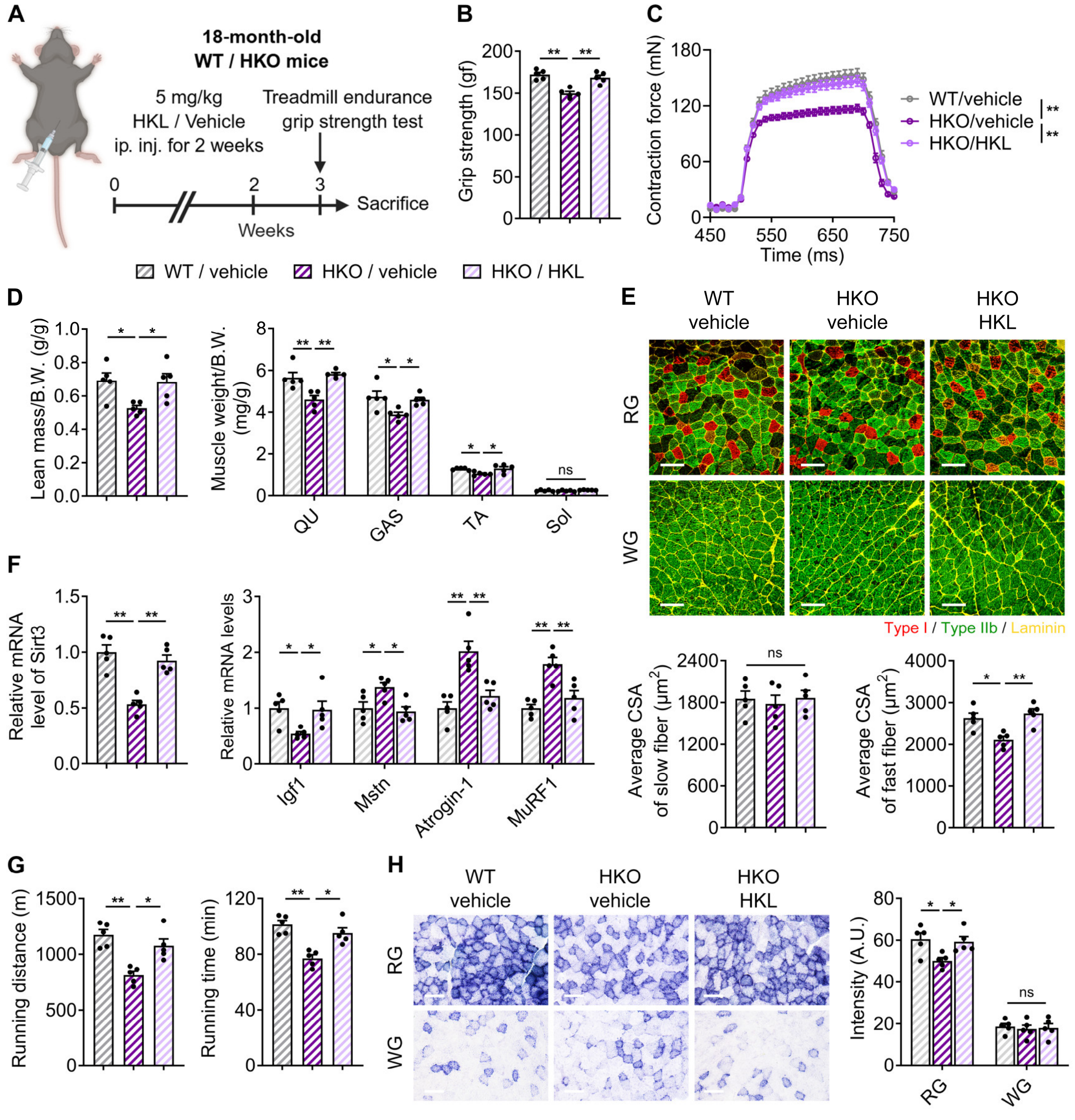

Figure S10. Pharmacological activation of SIRT3 alleviates muscle atrophy and mitochondrial dysfunction in HSF1 muscle-deficient mice

(A) Schematic illustration of SIRT3 pharmacological activation in aged (18-month-old) mice by intraperitoneal (i.p.) injection of Honokiol (HKL). (B) Grip strength and (C) tetanic contraction force of EDL muscle of the indicated groups. (D) Normalized lean mass and muscle weights of the indicated groups. (E) Representative MyHC IF staining in GAS muscles and quantification of fiber size of slow- and fast-twitch fibers of the indicated groups. (F) mRNA levels of *Sirt3* and atrophic genes in GAS muscles of the indicated groups. (G) Treadmill endurance performance of the indicated groups. (H) Representative SDH staining and quantification of SDH intensity of the indicated groups. Scale bar represents 100 μm. n=5 mice per group unless otherwise noted.

Statistical significance was assessed by one-way ANOVA with Tukey's multiple comparisons (B and D-H) and two-way ANOVA with Bonferroni's post hoc test (C). Data are presented as mean ± SEM and *$P<0.05$; **$P<0.01$; ns, not significant compared to control group.

Figure S11

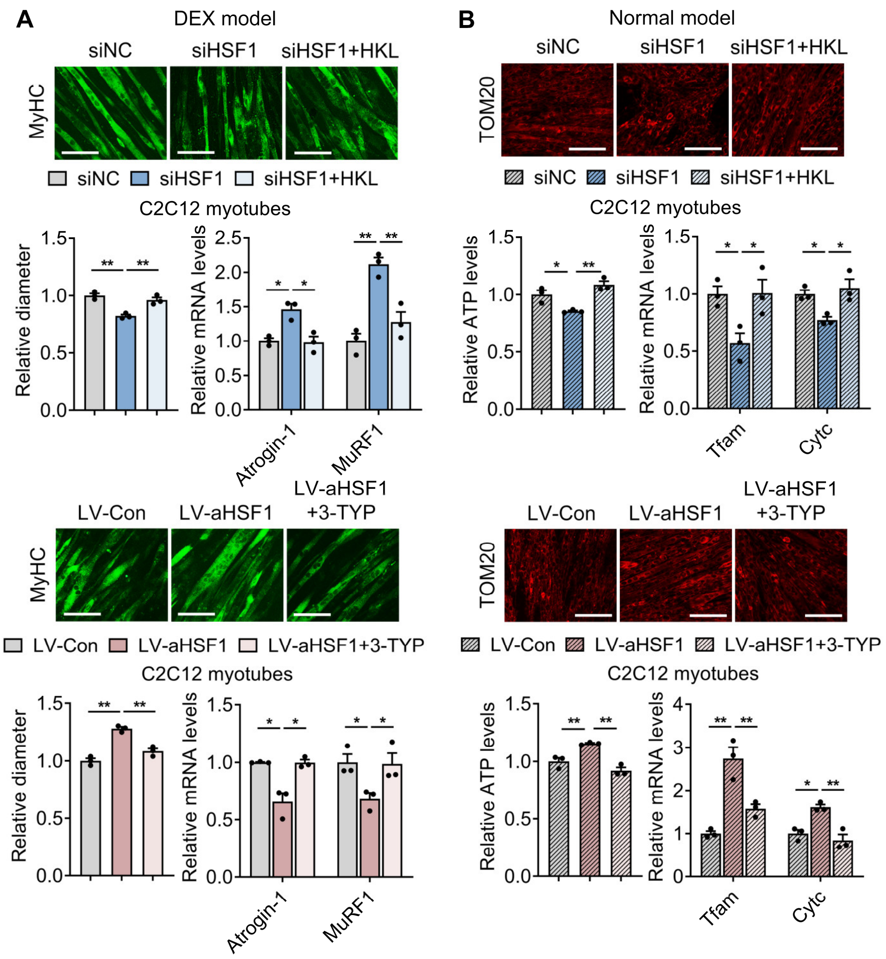

Figure S11. HSF1-SIRT3 axis ameliorates myotube atrophy and mitochondrial dysfunction *in vitro*

(A and B) Rescue experiments in two distinct C2C12 myotube models. (A) C2C12 myotubes were pretreated with DEX or vehicle for 24 hours, followed by HSF1 deficiency by siRNA or activation by lentivirus for an additional 24 hours, and SIRT3 activation with HKL or inhibitor with 3-TYP for another 24 hours. The top panels show representative images of myotubes. The bottom panel shows the quantification of myotube diameter and mRNA levels of atrophic genes in myotubes. (B) C2C12 myotubes were transfected with siHSF1 or LV-aHSF1 for HSF1 deficiency or activation without myotube atrophy, followed by SIRT3 activation or inhibitor for 24 hours. The top panels show representative images of mitochondria in myotubes. The bottom panel shows the ATP contents and mRNA levels of mitochondrial genes in myotubes. Scale bar represents 100 μm. n=3 per group.

Statistical significance was assessed by one-way ANOVA with Tukey's multiple comparisons (A and B). Data are presented as mean ± SEM and *P<0.05; **P<0.01; ns, not significant compared to control group.

Figure S12

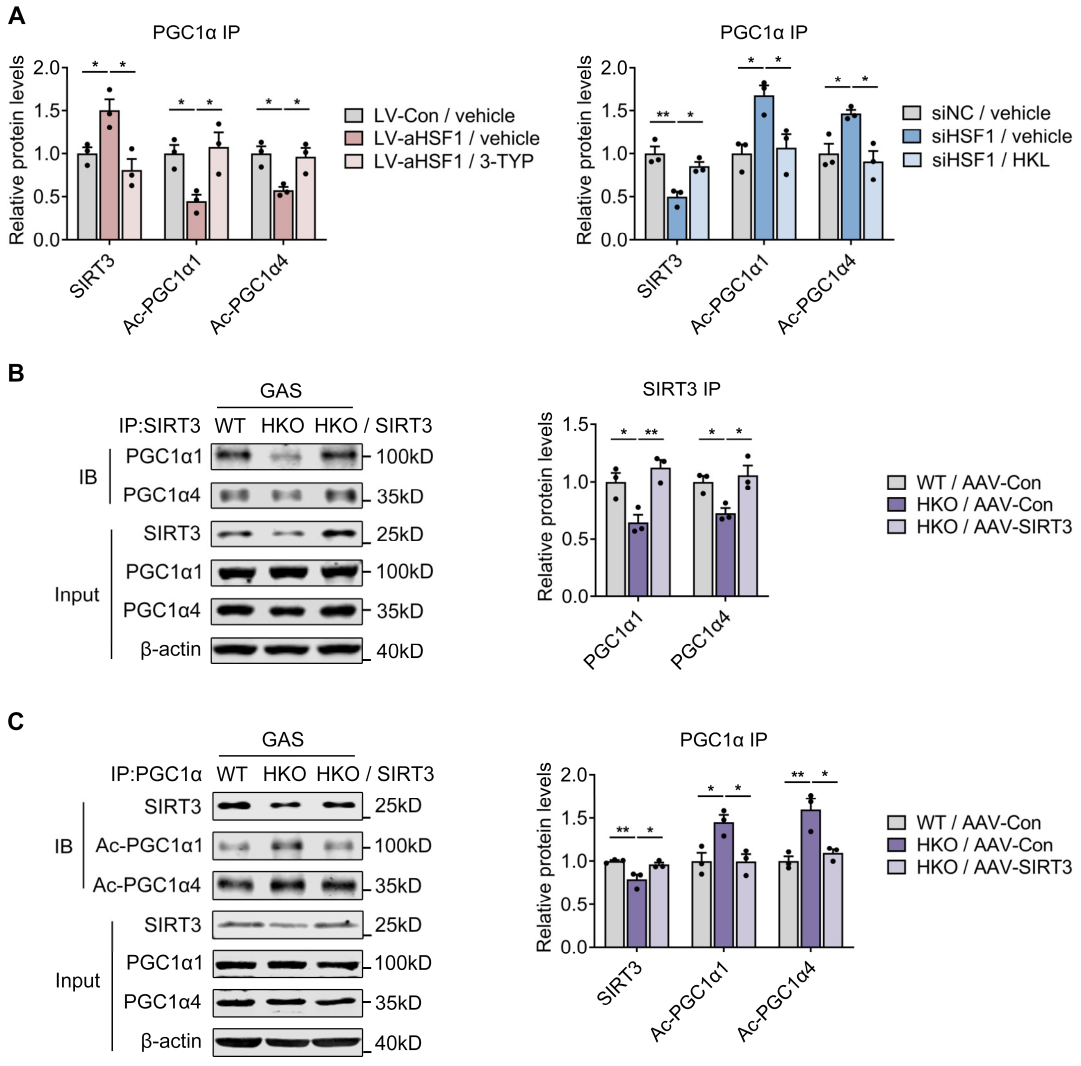

Figure S12. SIRT3 binds to and deacetylates PGC1α isoforms *in vitro* and *in vivo*

(A) Quantification of three independent endogenous co-IP experiments in C2C12 myotubes (related to Figure 8B). (B) Analysis of SIRT3-PGC1α interaction in GAS muscles shows representative immunoblot and quantification of PGC1α levels following co-IP with SIRT3 antibody. n=3 independent experiments. (C) Analysis of PGC1α acetylation in GAS muscles presents representative immunoblot and quantification of SIRT3 and PGC1α acetyl-lysine (Ac-Lys) levels following co-IP with PGC1α antibody. n=3 independent experiments.

Statistical significance was assessed by one-way ANOVA with Tukey's multiple comparisons (A-C). Data are presented as mean ± SEM and **P*<0.05; ***P*<0.01 compared to control group.

Figure S13

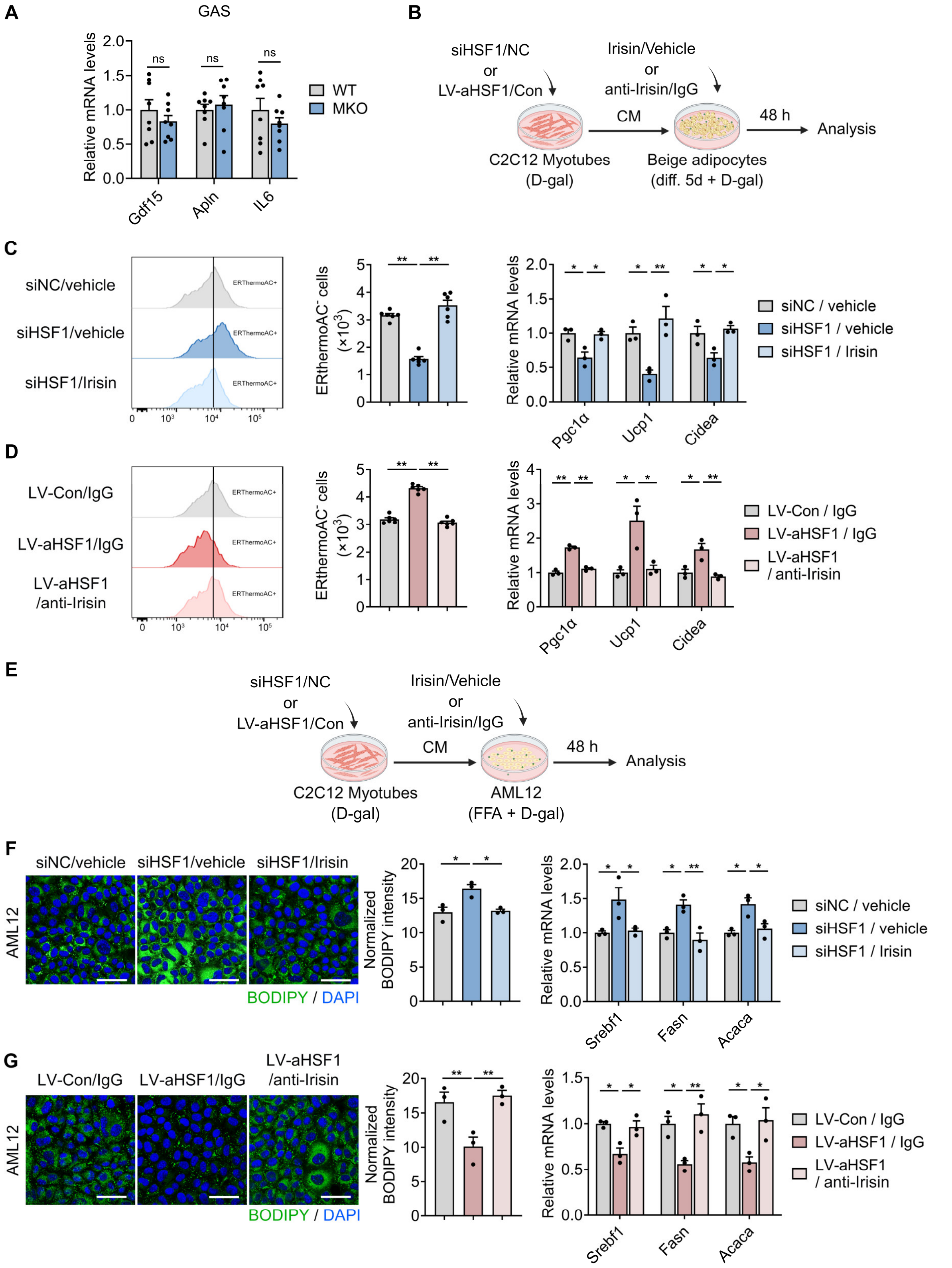

Figure S13. Irisin-mediated crosstalk between C2C12 myotubes and beige adipocytes or hepatocytes.

(A) mRNA levels of myokines and mitokines in GAS muscle of the indicated groups (n=8). (B-D) An *in vitro* muscle-adipose crosstalk model. (B) Schematic illustration of experimental procedures of crosstalk between C2C12 myotubes and differentiated beige adipocytes, which were both pretreated with D-gal to induce cellular senescence. (C-D) Flow cytometry analysis of the thermosensitive fluorescent dye stained ERthermAC-positive cells in C3H10T1/2-derived beige adipocytes treated with conditioned medium (CM) from the indicated groups (n=6), and mRNA levels of thermogenic genes of the indicated groups. (E-G) An *in vitro* muscle-liver crosstalk model. (E) Schematic illustration of experimental procedures of crosstalk between C2C12 myotubes and AML12 hepatocytes, which were both pretreated with D-gal to induce cellular senescence. (F-G) Representative BODIPY staining and quantification of lipid droplets in AML12 hepatocytes treated with CM from the indicated groups, and mRNA levels of hepatic de novo lipogenesis (DNL) genes of the indicated groups. Scale bar represents 50 μm. n=3 per group unless otherwise noted.

Statistical significance was assessed by unpaired Student's t test (A) and one-way ANOVA with Tukey's multiple comparisons (C, D, F and G). Data are presented as mean ± SEM and *$P<0.05$; **$P<0.01$ compared to control group.

Figure S14

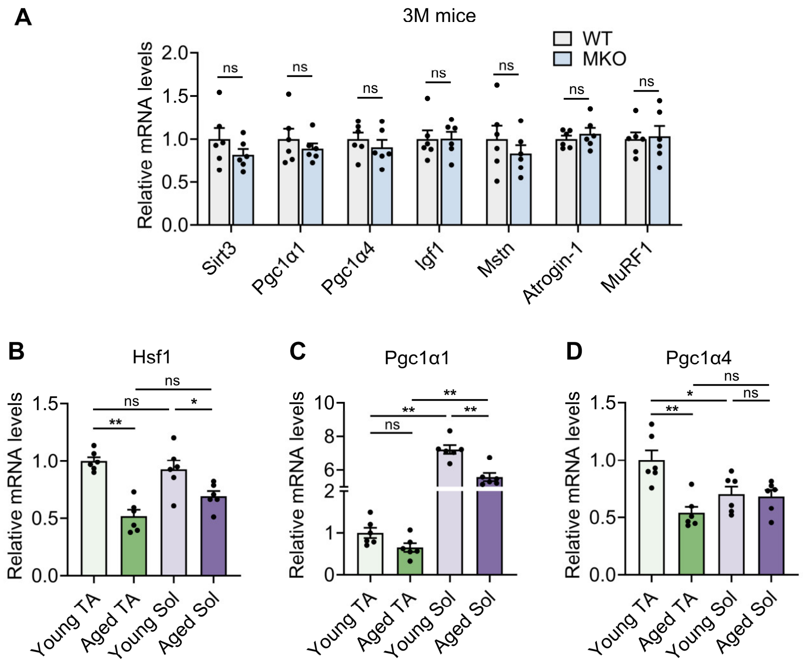

Figure S14. Differential expression patterns of HSF1 and PGC1α isoforms on specific fiber types in aging.

(A) mRNA levels of HSF1 target genes in young GAS muscles of the indicated genotypes. (B) mRNA levels of *Hsf1* in TA and Sol muscles of the indicated groups. (C) mRNA levels of *Pgc1α1* in TA and Sol muscles of the indicated groups. (D) mRNA levels of *Pgc1α4* in TA and Sol muscles of the indicated groups. n=6 mice per group unless otherwise noted.

Statistical significance was assessed by unpaired Student's t test (A) and one-way ANOVA with Tukey's multiple comparisons (B-D). Data are presented as mean ± SEM and **P*<0.05; ***P*<0.01; ns, not significant compared to control group.

Table S1. siRNA used in transfection

Name	Target sequence (5′→3′)	
siHsf1	Sense	GGAACAGCUUCCACGUGUUTT
	Antisense	AACACGUGGAAGCUGUUCCTT

Table S2. PCR primers used for the construction of SIRT3 promoters

Promoter	Primer sequence (5′→3′)	
SIRT3 full length & mutation	F1	CGGGGTACCCCGAGACAGGGTTTCTCT
	F2	TTCAGATGATGACAAAGAAGGCTTCTGGTG
	R1	CCGCTCGAGAGTAGGCGGGTGATAACT
	R2	CACCAGAAGCCTTCTTTGTCATCATCTGAA

Table S3. The sequences of primers for qPCR analysis

Gene name	Primer sequence (5′→3′)	
hHSF1	Forward	TGAAAAGTGCCTCAGCGTAGCC
	Reverse	TGCTCAGCATGGTCTGCAGGTT
hGAPDH	Forward	AAGACGGGCGGAGAGAAACC
	Reverse	CGTTGACTCCGACCTTCACC
mHsf1	Forward	CCGAAAAGTAGTCCACATTGAGC
	Reverse	GCTGGTCACTTTCCTCTTGATGT
mAtrogin-1	Forward	CAGCTTCGTGAGCGACCTC
	Reverse	GGCAGTCGAGAAGTCCAGTC
mMuRF1	Forward	TACCAAGCCTGTGGTCATCCTG
	Reverse	ACGGAAACGACCTCCAGACATG
mMstn	Forward	ACGAGTGGATGGTGCGCTGTGTGC
	Reverse	TCATTCTGAACGCGCATGAAGCG
mIgf1	Forward	CCACACTGACATGCCCAAGA
	Reverse	CCTGCACTTCCTCTACTTGTGTTC
mTfam	Forward	GAGGCAAAGGATGATTCGGCTC
	Reverse	CGAATCCTATCATCTTTAGCAAGC
mCytc	Forward	AAATCTCCACGGTCTGTTCGG
	Reverse	GGGTATCCTCTCCCCAGGTG
mUcp1	Forward	ACTGCCACACCTCCAGTCATT

	Reverse	CTTTGCCTCACTCAGGATTGG
mElovl3	Forward	TTCTCACGCGGGTTAAAAATGG
	Reverse	TCTCGAAGTCATAGGGTTGCAT
mCidea	Forward	TGACATTCATGGGATTGCAGAC
	Reverse	CGAGCTGGATGTATGAGGGG
mPgc1α	Forward	AGCCGTGACCACTGACAACGAG
	Reverse	GCTGCATGGTTCTGAGTGCTAAG
mCpt1α	Forward	AGATCAATCGGACCCTAGACAC
	Reverse	CAGCGAGTAGCGCATAGTCA
mMcad	Forward	TCCTAAAGCTCCTGCTAATAAAGC
	Reverse	ATCGCTGGCCCATGTTTAAT
mCd36	Forward	TTTGGAGTGGTAGTAAAAAGGGC
	Reverse	TGACATCAGGGACTCAGAGTAG
mApoe	Forward	CTGACAGGATGCCTAGCCG
	Reverse	CGCAGGTAATCCCAGAAGC
mPparα	Forward	AACATCGAGTGTCGAATATGTGG
	Reverse	CCGAATAGTTCGCCGAAAGAA
mPgc1α1	Forward	GGACATGTGCAGCCAAGACTCT
	Reverse	CACTTCAATCCACCCAGAAAGCT
mPgc1α4	Forward	TCACACCAAACCCACAGAAA
	Reverse	CTGGAAGATATGGCACAT
mFndc5	Forward	ATGAAGGAGATGGGGAGGAA
	Reverse	GCGGCAGAAGAGAGCTATAACA
mMyh7	Forward	GCCAACTATGCTGGAGCTGATGCCC
	Reverse	GGTGCGTGGAGCGCAAGTTTGTCATAAG
mMyh2	Forward	AAGTGACTGTGAAAACAGAAGCA
	Reverse	GCAGCCATTTGTAAGGGTTGAC
mMyh4	Forward	CTTTGCTTACGTCAGTCAAGGT
	Reverse	AGCGCCTGTGAGCTTGTAAA
mMyh1	Forward	GGCAGCAGCAGCTGCGGAAGCAGAGTCTGG
	Reverse	GAGTGCTCCTCAGATTGGTCATTAGC
mTnfα	Forward	ATGGCCTCCCTCTCATCAGT
	Reverse	TTTGCTACGACGTGGGCTAC

mIL1β	Forward	GCAACTGTTCCTGAACTCAACT
	Reverse	ATCTTTTGGGGTCCGTCAACT
mIL6	Forward	TACCACTTCACAAGTCGGAGGC
	Reverse	CTGCAAGTGCATCATCGTTGTTC
mSrebf1	Forward	GAAGCTGTCGGGGTAGCGTCT
	Reverse	CTCTCAGGAGAGTTGGCACCTG
mFasn	Forward	GGAGGTGGTGATAGCCGGTAT
	Reverse	TGGGTAATCCATAGAGCCCAG
mAcaca	Forward	ACACCATGTTGGGAGTTGTG
	Reverse	GCTGTTCCTCAGGCTCACAT
mTgfβ	Forward	TGATACGCCTGAGTGGCTGTCT
	Reverse	CACAAGAGCAGTGAGCGCTGAA
mDrp1	Forward	CCTCAGATCGTCGTAGTGGGA
	Reverse	GTTCCTCTGGGAAGAAGGTCC
mFis1	Forward	AGAGCACGCAATTTGAATATGCC
	Reverse	ATAGTCCCGCTGTTCCTCTTT
mMief1	Forward	GTACTTGAGTGGCAGCCTCTATG
	Reverse	CCAGGAAGAAACCAGGGACATTC
mMief2	Forward	GGACTTCCTCTTGGCTAATGCTC
	Reverse	CCTTGGTGTCATCCTCATCTGG
mOpa1	Forward	TGGAAAATGGTTCGAGAGTCAG
	Reverse	CATTCCGTCTCTAGGTTAAAGCG
mMfn1	Forward	ATGGCAGAAACGGTATCTCCA
	Reverse	GCCCTCAGTAACAAACTCCAGT
mMfn2	Forward	AGAACTGGACCCGGTTACCA
	Reverse	CACTTCGCTGATACCCCTGA
mFst	Forward	GCCAGTGACAATGCCACATACG
	Reverse	CTTCCTCCGTTTCTTCCGAGATG
mWisp	Forward	AGGAAAGTCGCCTCTGCAACCT
	Reverse	TGACACAGCCTGCGAGAGTGAA
mErrα	Forward	ACTACGGTGTGGCATCCTGTGA
	Reverse	GGTGATCTCACACTCATTGGAGG
mGdf15	Forward	AGCCGAGAGGACTCGAACTCAG

	Reverse	GGTTGACGCGGAGTAGCAGCT
mApln	Forward	AGGCATAGCGTCCTCACCTCTT
	Reverse	GGTGCAGAAACGACAAAGACGG
mGapdh	Forward	ACAACTTTGGCATTGTGGAA
	Reverse	GATGCAGGGATGATGTTCTG
m36B4	Forward	AGATTCGGGATATGCTGTTGGC
	Reverse	TCGGGTCCTAGACCAGTGTTC

Table S4. Primers used in ChIP assays

Target name	Target sequence (5′→3′)	
SIRT3-HSE	Forward	TCAGATGATGACAAGAAAGTTCCA
	Reverse	AAAACACACTCTTTCCCATGCT
β-globin	Forward	AAGCCTGATTCCGTAGAGCCACAC
	Reverse	CCCACAGGCA AGAGACAGCAGC
